# The association between women's sanitation experiences and mental health: A cross-sectional study in Rural, Odisha India

**DOI:** 10.1016/j.ssmph.2018.06.005

**Published:** 2018-06-20

**Authors:** Bethany A. Caruso, Hannah L.F. Cooper, Regine Haardörfer, Kathryn M. Yount, Parimita Routray, Belen Torondel, Thomas Clasen

**Affiliations:** a Department of Behavioral Sciences and Health Education, Rollins School of Public Health, Emory University, Atlanta, GA, USA; b Department of Environmental Health, Rollins School of Public Health, Emory University, Atlanta, GA, USA; c Hubert Department of Global Health, Rollins School of Public Health, Emory University, Atlanta, GA, USA; d Department of Sociology, Emory University, Atlanta, GA, USA; e Department of Disease Control, London School of Hygiene and Tropical Medicine, London, UK

**Keywords:** Mental health, Well-being, Urination, Defecation, Gender, Life course

## Abstract

Emerging qualitative research suggests women’s sanitation experiences may impact mental health. However, specific associations remain unclear. We aimed to determine if sanitation access and sanitation experiences were associated with mental health among women in rural Odisha, India. Using a cross-sectional design, we evaluated the association between sanitation access and sanitation experiences and selected mental health outcomes. Data were collected from 1347 randomly selected women across four life course stages in 60 rural communities (December 2014-February 2015). Our four primary outcomes included: mental well-being, and symptoms of anxiety, depression, and distress. The primary exposures were (1) access to a functional latrine within the household compound and (2) sanitation insecurity (SI), evaluated using a seven domain measure assessing women’s negative sanitation experiences and concerns. We used hierarchical linear modeling to determine associations between the exposures and mental health outcomes, adjusting for covariates (life stage, poverty, current health status, social support). Mean well-being scores were moderate and mean anxiety, depression, and distress scores were above a threshold indicating the potential presence of any of the three conditions. Access to a functional household latrine was associated with higher well-being scores, but not with anxiety, depression or distress. Women’s SI domains were associated with all four outcomes: four domains were significantly associated with lower well-being scores, two were significantly associated with higher anxiety scores, three were significantly associated with higher depression scores, and three were significantly associated with higher distress scores, all independent of functional household latrine access. Women in rural Odisha, India may suffer assaults to their well-being and have higher symptoms of anxiety, depression, and distress when urinating and defecating, even if they have an available facility. These findings suggest that sanitation-related interventions should consider how to accommodate women’s experiences beyond excreta management to comprehensively impact health.

## Highlights

• Well-being, anxiety, depression, and distress scores were modest to poor overall.
• Functional household latrine access was associated with higher well being scores.
• Access was not associated with anxiety, depression, or distress.
• Women’s sanitation insecurity domains were associated with all four outcomes.
• Sanitation experiences may impact mental health even among latrine owners.

## 1 Introduction

An estimated 2.3 billion people lack access to basic sanitation, an unshared household facility hygienically separating human excreta from human contact. Among these, an estimated 892 million lack access to any kind of sanitation facility and practice open defecation (JMP, 2017). The effects of improved sanitation on infectious disease are substantial; eliminating exposure to human feces reduces risk of diarrhea, trachoma, schistosomiasis, and soil-transmitted helminthes, which can result in stunting, cognitive impairment, tropical enteropathy, or death, particularly among children under age five (Berkman, Lescano, Gilman, Lopez, & Black, 2002; Dillingham & Guerrant, 2004; Freeman et al., 2017; Grimes et al., 2014; Guerrant, DeBoer, Moore, Scharf, & Lima, 2013; Stocks et al., 2014; Wolf et al., 2014; Ziegelbauer et al., 2012). While infectious disease health outcomes are critical, the World Health Organization defines health more broadly as “a state of complete physical, mental and social well-being and not merely the absence of disease or infirmity”(WHO, 1946). Despite calls for broader investigations of sanitation-related health impacts (Caruso, Sevilimedu, Fung, Patkar, & Baker, 2015; Pradyumna, Prahlad, & Ganesh, 2015), research beyond infectious disease is limited.

Women may be particularly at risk for non-infectious disease outcomes if they lack access to sanitation environments that accommodate their needs. Poor sanitation has been associated with maternal mortality (Benova, Cumming, & Campbell, 2014), and open defecation with increased odds of adverse pregnancy outcomes (Padhi et al., 2015) and non-partner violence (Jadhav, Weitzman, & Smith-Greenaway, 2016; Winter & Barchi, 2016). Increasingly, research has documented the health risks of women’s sanitation experiences. Women have reported shame if seen by others, withholding food and water to limit urination or defecation, suppressing needs due to inhospitable physical or social environments, being unable to tend to needs due to obligations, fearing or experiencing physical or sexual violence when accessing locations or addressing needs, and feeling helplessness to change sanitation conditions (Caruso, Clasen, Hadley, et al., 2017; Joshi, Fawcett, & Mannan, 2011; Kulkarni, O’Reilly, & Bhat, 2017; O’Reilly, 2016; Routray, Torondel, Clasen, & Schmidt, 2017).

Qualitative research suggests that sanitation-related experiences may lead to increased psychosocial stress among women. Factors concerning the physical and social environments, personal constraints, safety, sexual violence, and finance have all been reported to contribute to women’s experiences of stress from sanitation (Bisung and Elliott, 2016a, Bisung and Elliott, 2016b; Hirve et al., 2015; O’Reilly, 2016; Sahoo et al., 2015). Research in India found that life stage and geographic location contributed to the perceived severity of sanitation-related physical, social, and sexual violence stressors reported (Hulland et al., 2015). These studies illuminate women’s experiences, but have limited ability to demonstrate if and how sanitation access and experiences may be associated with mental health outcomes.

While associations with sanitation experiences and mental health outcomes have not been assessed previously, researchers have found associations between women’s experiences of water and psychosocial distress and anxiety among women in Brazil, Bolivia, and Ethiopia. Coêlho, Adair, and Mocellin (2017) found significantly higher levels of anxiety and emotional distress among participants in a drought prone area compared to those in a drought free area in Brazil and they found women to be more emotionally distressed and anxious than men. From their research in Bolivia, Wutich and Ragsdale (2008) found water insecurity to be associated with emotional distress and that women experienced more emotional distress related to water than did men. And in Ethiopia, Stevenson et al. (2012) found water insecurity (evaluated using their created measure) to be significantly associated with distress. This research on the lived experience of water lays the groundwork for research to move beyond qualitative assessments and to determine if the lived experiences of sanitation may be associated with mental health outcomes.

We aimed to determine quantitatively if sanitation is associated with mental well-being, and symptoms of anxiety, depression, and distress among women in rural Odisha, India. We evaluated two sanitation-related exposures: access to a functional household latrine and “Sanitation Insecurity” (SI), a measure created from the voiced concerns of women to assess the frequency of their negative sanitation experiences (Caruso, Clasen, Yount, et al., 2017; Caruso, Clasen, Hadley, et al., 2017). Because women have varied experiences and needs at different life stages (Bustreo et al., 2013; Parakh, 2011), we incorporate life stage into our model to determine influence.

## 2 Methods

### 2.1 Setting and study design

We conducted a cross-sectional study to evaluate the association between the sanitation exposures and selected mental health outcomes. Data were collected from December 2014-February 2015 in rural communities of Odisha, India, where open defecation is the norm (Routray, Schmidt, Boisson, Clasen, & Jenkins, 2015). Our study took place in communities that previously participated in a cluster randomized controlled trial (CRT) designed to assess the impact of a sanitation intervention on diarrhea, soil-transmitted helminth infection, and child malnutrition (Clasen et al., 2012, Clasen et al., 2014; Boisson et al., 2014). The intervention did not result in improvements in any of the health outcomes.

### 2.2 Target sample size

A simulation study informed sample size. This simulation demonstrated power to detect 20% direct and cross-level interaction effects using multilevel (hierarchical) modeling for a continuous level-2 predictor to be greater than 96% for 60 clusters of 20 participants (Estes, 2008). Power was sufficient for both continuous and dichotomous predictors in a base sample size of 1200. We aimed to sample 1440 individuals (24 per community) anticipating 20% non-response due to 1) incomplete surveys or 2) sampling error (i.e. ineligibility, misclassification).

### 2.3 Sampling procedure

We used a stratified, multi-stage, cluster sample design. We sampled two units: communities and women living in these communities. We identified 60 communities from the 100 that were engaged in the aforementioned CRT, 30 former intervention and 30 former control, to determine influence of previous intervention status on outcomes. To be eligible, former intervention communities needed to have latrine coverage greater than 25%, and former control communities needed to have latrine coverage less than 20%. These sanitation cut-points were intended to serve as proxies for moderate and poor coverage. We used endline data from the trial (December 2014) to select former intervention communities, assuming little coverage change. For former control communities, we sought data from a non-government organization (NGO) partner actively working to provide sanitation in these communities. Communities were ineligible if members had participated in qualitative research that informed the current study. Thirty-one communities in each arm met our eligibility criteria; we selected the 30 intervention communities with the greatest coverage and the 30 control communities with the least coverage.

We sought to recruit 24 women living in each community, with variation in the sample by life stage given reported differences in sanitation experiences (Caruso, Clasen, Yount, et al., 2017; Caruso, Clasen, Hadley, et al., 2017; Hulland et al., 2015; Sahoo et al., 2015). To create a sampling frame, we conducted a census in each selected community to identify women over 18 years of age in four life stages: (1) unmarried, age 49 or younger (2) married three years or less, (3) married over three years and age 49 or younger, and (4) over 49 years of age of any marital status. These life stage categories were informed by previous research and literature and we suspected they would influence the outcomes of interest. Specifically, unmarried women typically live in the home of their parents and exhibit greater control of resources than recently married women, who generally have restricted mobility (Joshi et al., 2011, Routray et al., 2015). Compared to recently married women, women married longer have both greater social status and freedom of movement around the community (Medhi, 2013). Older women are under-represented in sanitation studies and are not included in national-level surveys that include women under age 49 only. However, due to their aging status, their quality of life may be influenced by increased incontinence risk and difficulty walking or squatting among other factors (Singh et al., 2013). We generated four sampling lists per community, one for each life stage category. Women were eligible if they were randomly selected from a list.

### 2.4 Data collection

Enumerators sought to survey six women per life stage category in each community. They skipped eligible participants if someone in the household already participated. All surveys were conducted in Oriya, the local language. Responses were recorded using pen and paper by eleven trained female enumerators. Data were double entered; all inconsistencies were checked against surveys and corrected (See S1 Text for additional data collection information).

### 2.5 Measures

#### 2.5.1 Outcomes

We selected four outcomes— subjective mental well-being, anxiety, depression, and distress—because each assess a different facet of mental health.

We used the World Health Organization Well-being Index (WHO-5) to measure subjective mental well-being (Bech, 2004). Well-being is generally characterized by the presence of positive emotions, the absence of negative emotions, being satisfied with life, judging life positively, and feeling good (Diener and Chan, 2011; Ryff and Keyes, 1995; Veenhoven, 2008). Well-being has been associated with longevity, quicker recovery from illness, lowered perception of pain, and protection against cardiovascular disease risk (Diener and Chan, 2011; Fredrickson, 2000; Pressman and Cohen, 2005) and is an important health state unto itself. The WHO-5 has adequate validity in research to evaluate differences between populations (Topp, Ostergaard, Sondergaard, & Bech, 2015). It consists of five statements (e.g. ‘I have felt cheerful and in good spirits’). Participants indicate how frequently they have related to each in the previous two weeks, from ‘At no time’ (1) to ‘All of the time’ (5)). Scores can range 0–25. The higher the score, the better the well-being; scores below 13 indicate poor well-being (Cronbach’s alpha from this sample = 0.88).

We used the Hopkins Symptoms Checklist (HSCL) to assess symptoms of anxiety, depression, and non-specific emotional distress (Derogatis, Lipman, Rickels, Uhlenhuth, & Covi, 1974). Anxiety is characterized by temporary worry or fear, and is normal to experience; anxiety disorders involve worry and fear that does not subside and may get worse (NIMH, 2015). Depression, characterized by low mood, loss of interest in previously enjoyable activities, and guilt, is an effective disorder that can evolve into a chronic condition or lead to suicide if untreated. It is associated with unhealthy behaviors like physical inactivity, poor diet, drinking, smoking, and sleep disorders that can lead to other illnesses (Boden and Fergusson, 2011; Dierker, Avenevoli, Stolar, & Merikangas, 2014; Lopresti, Hood, & Drummond, 2013). Distress is a broad category that includes symptoms related to anxiety, depression, and adjustment disorder (Bidstrup et al., 2015).

Used in India and other global settings (Kaaya et al., 2002, Mollica et al., 1987, Weaver and Kaiser, 2015), the HSCL consists of 25 symptoms and asks respondents to indicate how much the symptoms bothered them in the prior week (‘Not at all’(1) to ‘Extremely’(4)). The first ten checklist symptoms assess anxiety, the final 15 assess depression, and the 25 collectively assess non-specific emotional distress. For each, the score is the mean. Scores greater than 1.75 indicate the potential presence of the condition; the lower the score the less anxiety, depression, or distress (Derogatis et al., 1974). While the HSCL is not diagnostic, we assume that greater endorsement of symptoms indicates a poorer condition. We omitted two items from the depression set. An item on sexual desire was deemed inappropriate for unmarried women. Another item on suicide ideation was deleted as we had no ability to provide clinical recourse if needed. The final tool included 10 items for anxiety (Cronbach’s alpha for our sample = 0.81), 13 for depression (Cronbach’s alpha from this sample = 0.86), and 23 items for non-specific emotional distress (Cronbach’s alpha from this sample = 0.90).

The WHO5 and HSCL were translated, back translated, and piloted extensively prior to use.

#### 2.5.2 Primary exposures

Primary exposures were access to a functional household latrine and *Sanitation Insecurity*. To assess access, we asked participants if they had a latrine and if it was functional. We determined participants to have access only if they responded yes to both.

We assessed sanitation-related experiences using the 50-item sanitation insecurity measure, a validated measure developed in Odisha, India designed to assess urination and defecation concerns and experiences across seven domains: ‘Potential harms’ (perception of harms when urinating/defecating); ‘Social expectations and repercussions’ (social constraints experienced when urinating/defecating); ‘Physical exertion or strain’ (concerns/experiences related to physically managing basic defecation/urination needs); ‘Night concerns’ (fears when urinating/defecating at night); ‘Dependent support’ (concerns for dependents when urinating/defecating); ‘Physical agility’ (concerns related to falling, pain and difficulty when squatting to urinate/defecate); and ‘Defecation place’ (concerns related to defecation location) (Caruso, Clasen, Yount, et al., 2017).

Response options ranged from Never (0) to Always (3). Participants receive a score for each domain, which was the mean of domain responses. Scores align with the response options to facilitate interpretation (range 0–3). Higher values indicated greater frequency of having experiences associated with the domain (Cronbach’s alphas reported in Table 1).

**Table 1:** Demographic characteristics of survey participants, overall and by life stage in rural Orissa, India (N = 1347).

	**All**		**1. Unmarried (UM)**		**2. Recently Married (RM)**		**3. Married (M)**		**4. Over 49 (OW)**	
**Number of Participants**	1347		328	24.4%	301	22.3%	376	27.9%	342	25.4%
**Former Intervention Community**	677	50.3%	163	49.7%	149	49.5%	194	51.6%	171	50.0%
**Age** (Range: 18–100)	36.6	(17.9)	21.2	(3.0)	23.9	(3.0)	35.4	(7.0)	63.7	(10.0)
**Hindu**	1329	98.7%	326	99.4%	296	98.3%	368	97.9%	339	99.1%
										
**Caste**^**1**^										
Brahmin	37	2.8%	10	3.1%	7	2.3%	12	3.2%	8	2.3%
General Caste	599	44.5%	146	44.6%	141	47.0%	162	43.1%	150	43.9%
Scheduled Caste (SC)	240	17.8%	50	15.3%	58	19.3%	73	19.4%	59	17.3%
Other Backward Caste (OBC)	439	32.6%	116	35.5%	85	28.3%	121	32.2%	117	34.2%
Scheduled Tribe (ST)	11	0.8%	2	0.6%	2	0.7%	3	0.8%	4	1.2%
Don’t Know	19	1.4%	3	0.9%	7	2.3%	5	1.3%	4	1.2%
										
**Education**										
None	323	24.0%	3	0.9%	6	2.0%	78	20.7%	236	69.0%
Some Primary	392	29.1%	51	15.5%	65	21.6%	178	47.3%	98	28.7%
Some Secondary	562	41.7%	228	69.5%	217	72.1%	109	29.0%	8	2.3%
Higher than Secondary	70	5.2%	46	14.0%	13	4.3%	11	2.9%	0	0.0%
**Below Poverty Line (BPL) Card**	889	66.0%	226	68.9%	192	63.8%	234	62.2%	237	69.3%
**Have Children**	874	64.9%	0	0.0%	173	57.5%	366	97.3%	335	98.0%
**Number of Children**	2.0	(2.2)	0	(0.0)	0.6	(0.6)	2.4	(1.2)	4.6	(2.2)
**No Current Illness**	1079	80.1%	298	90.9%	282	93.7%	313	83.2%	186	54.4%
**Social Support** (Potential and actual range: 0–4)	2.7	(1.0)	3.3	(0.9)	2.8	(1.0)	2.5	(0.9)	2.1	(0.8)
										
**Household Water and Sanitation Access**										
Functional Latrine in Household	483	35.9%	92	28.1%	143	47.5%	117	31.1%	131	38.3%
Primary Drinking Water Source within Dwelling/Compound	402	29.8%	82	25.0%	114	37.9%	102	27.1%	104	30.4%
Bathing Room in Household	204	15.1%	25	7.6%	85	28.2%	48	12.8%	46	13.5%
										
**Sanitation Insecurity Domains (potential score range: 0–3)**										
1: Potential Harms (Actual range: 0–3, Cronbach’s alpha = 0.90)	0.8	(0.8)	1.0	(0.8)	0.9	(0.8)	0.8	(0.7)	0.5	(0.6)
2: Social Expectations & Repercussions (Actual range: 0–2.2, Cronbach’s alpha = 0.86)	0.4	(0.4)	0.5	(0.5)	0.5	(0.5)	0.4	(0.4)	0.3	(0.3)
3: Physical Exertion / Strain (Actual range: 0–2.7, Cronbach’s alpha = 0.64)	0.1	(0.3)	0.1	(0.3)	0.1	(0.3)	0.1	(0.2)	0.1	(0.2)
4: Night Concerns (Actual range: 0–3, Cronbach’s alpha = 0.91)	1.2	(1.1)	1.6	(1.1)	1.5	(1.1)	1.1	(1.0)	0.7	(0.9)
5: Social Support (Actual range: 0–3, Cronbach’s alpha = 0.89)	0.2	(0.4)	0.0	(0.2)	0.4	(0.7)	0.2	(0.4)	0.0	(0.1)
6: Physical Agility (Actual range: 0–3, Cronbach’s alpha = 0.81)	0.5	(0.8)	0.2	(0.4)	0.3	(0.6)	0.3	(0.6)	1.1	(1.0)
7: Defecation Place (Actual range: 0–3, Cronbach’s alpha = 0.90)	1.1	(0.9)	1.3	(0.9)	1.0	(1.0)	1.2	(0.9)	1.0	(0.8)
										
**Mental Health Outcomes**										
WHO5 Well-Being (Potential and actual range: 0–25)	13.9	(7.5)	16.6	(6.9)	15.8	(6.9)	13.3	(7.3)	10.1	(7.0)
HSCL Anxiety (Potential and actual range 1–4)	1.9	(0.6)	1.8	(0.6)	1.9	(0.7)	1.8	(0.6)	2.0	(0.6)
HSCL Depression (Potential and actual range 1–4)	1.8	(0.6)	1.7	(0.6)	1.8	(0.6)	1.8	(0.6)	2.1	(0.6)
HSCL Non-Specific Emotional Distress (Potential range: 1–4, actual range: 1–3.8)	1.8	(0.6)	1.7	(0.5)	1.8	(0.6)	1.8	(0.5)	2.0	(0.6)

Data are number and percent or mean and (standard deviation).

1. For *Caste:* 2 missing, one from stage 1 and one from stage 2.

#### 2.5.3 Covariates

We included individual-level covariates that have been found to influence mental health outcomes, including: life stage, economic status (assessed by ownership of a ‘Below the Poverty Line’ (BPL) card enabling government support), current health status, and perceived social support (Parakh, 2011; Patel, 2005). We included two covariates that are linked to sanitation behavior: access to water within the household compound and access to a room for bathing (typically an outside room without direct water access). Previous research in Odisha used BPL card possession as a proxy for economic status (Clasen et al., 2014). We assessed perceived social support using the Multidimensional Scale for Perceived Social Support (Zimet, Dahlem, Zimet, & Farley, 1988). The 12-item scale assesses perceived social support from family, friends, and a significant other. Informed by Mohanty, Ahn, and Chokkanathan (2014), we only used the 8 items representing family and friends because unmarried women were not likely to have a significant other. Scale response options ranged from completely disagree to completely agree (0–4); final scores were the mean and align with the response options (Cronbach’s alpha from this sample = 0.85). We assessed previous intervention status at the community-level.

### 2.6 Analysis

For each outcome, we estimated five successive hierarchical linear models using maximum likelihood estimation to model clustering of individual women (Level 1, L1) within communities (Level 2, L2). We specifically elected to use hierarchical linear modeling because we expected the intervention status of the community from the preceding trial, specifically whether or not it received the intervention or served as a control, may have in impact on our outcomes of interest. In model 1, we estimated an unconditional model to determine the intraclass correlation coefficient (ICC), the proportion of variance that can be explained by the communities (clusters) (McCoach, 2010). In model 2, we ran a multilevel bivariate model that regressed the outcomes on latrine ownership. In model 3, we created a model with latrine ownership and sanitation insecurity to determine if sanitation insecurity was associated with the outcomes, accounting for latrine ownership. In model 4, we added all individual-level covariates. In model 5, we added intervention status, the cluster (community) level covariate, to determine influence of previous trial status.

For each outcome, we calculated the proportional reduction in variance and the proportional reduction in prediction error for each successive model, comparing each model to the prior, more parsimonious model (Raudenbush and Bryk, 2002). To include the same participants in each outcome modeled, we excluded 62 participants with missing predictor or outcome data (4% of overall sample).

We used SAS (Cary, NC, USA; version 9.3) to generate descriptive statistics and HLM Software (Skokie, IL, USA; version 7.1) for hierarchical linear models.

### 2.7 Ethics

The Institutional Review Board at Emory University (Atlanta, GA, USA; IRB00072840) and the Institutional Ethics Committee of KIIT University (Bhubaneswar, India; KIMS/KIIT/IEC/795/2014) provided ethical approval of this study. Participants provided oral consent prior to participation. We report as per STROBE guidelines (See Supplemental Text 2).

## 3 Results

### 3.1 Sample size and socio-demographic characteristics

**Fig. 1:**
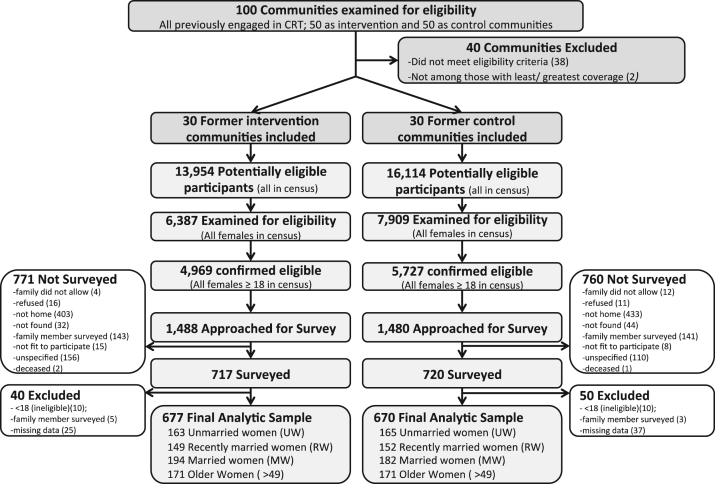
Flow diagram illustrating community and individual eligibility, exclusion, non-participation, and inclusion in final analysis.

In the 60 communities engaged (mean population: 540 former control, 465 former intervention), 1437 women were surveyed of 2968 approached (See Fig. 1). Ninety were excluded from analysis because of: missing outcome or predictor data (62), participation of another household member (20), or ineligibility (8). The final analytic sample consisted of 1347 participants, including 328 unmarried women (25%) (mean age: 21; range 18–39), 301 recently married women (22%) (mean age: 24; range 18–38), 376 women married over three years (28%) (mean age: 35; range 20–49), and 342 women over age 49 (25%) (mean age: 64; range 50–100).

Almost all women were Hindu (99%); 45% belonged to the general caste, meaning they did not receive caste-based government support; 66% had a BPL card; 80% indicated they were not suffering from a current illness; 30% reported access to a primary water source within the household dwelling/compound, and 15% reported access to a bathing room (Table 1).

Thirty-six percent reported access to a functional household latrine. Access to sanitation, water and bathing areas varied by life stage, with recently married women having the greatest access and unmarried women having the least. Mean scores for all seven sanitation insecurity domains were low overall, ranging from 0.1 (physical exertion /strain) to 1.2 (Night concerns). For each domain, scores were progressively lower along life stage categories and among women who had access to a latrine compared to women who did not (S1 Table).

### 3.2 Participant mental well-being, anxiety, depression and distress scores

**Fig. 2:**
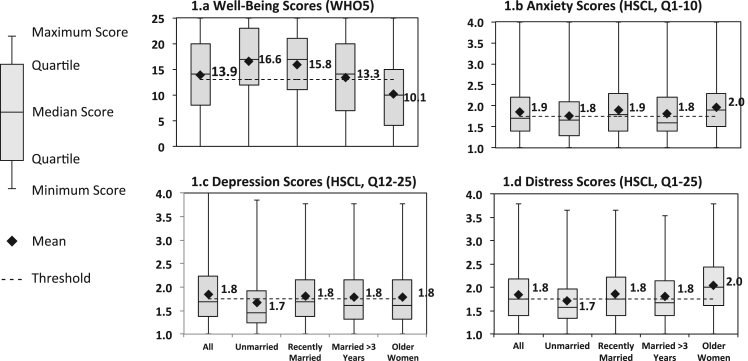
**Well-being, anxiety, depression, and distress scores among study participants in rural Orissa, India.** For well-being (a), scores below the threshold ( < 13) represent poor well-being. For anxiety (b), depression (c), and distress (d), scores above the threshold ( < 1.75) indicate the potential presence of the condition.

The overall scores for well-being (mean 13.9; Standard deviation (SD) 7.5; range: 0–25), anxiety (mean 1.9; SD 0.6; range: 1–4), depression (mean 1.8; SD 0.6; range: 1–4), and non-specific emotional distress (mean 1.8; SD 0.6; range: 1–4), were moderate overall. Scores were higher for well-being and lower for anxiety, depression, and distress at earlier life stages compared to later life stages (Fig. 1). Well-being scores were negatively, but not strongly correlated, with scores of the other outcomes. Anxiety, depression, and distress scores were strongly correlated (S2 Table) Fig. 2.

### 3.3 Multivariate results

For all outcomes, ICCs were low (range 0.05–0.08) indicating very little heterogeneity between the communities (See S3, S4, S5, and S6 Tables for Models 1–5 for all outcomes).

#### 3.3.1 Well-being

In the full model, there was a positive association between functional household latrine access and well-being (β = 3.37, P < 0.001) (Table 2). There was a negative association between four domains of sanitation insecurity (‘Potential harms’, ‘Physical exertion or strain’, ‘Night concerns’, and ‘Physical Agility’) and well-being, despite latrine access. Illustratively, a one point increase in score for ‘Physical exertion or strain’ was associated with a 3.06 decrease in well-being score (p < 0.001). One of the sanitation insecurity domains (Domain 7: Defecation place) had a positive effect on well-being (β = 1.38, P = 0.005). There was a negative association of life stage on well-being; scores were progressively higher through the life stages compared with unmarried women, the referent group. There was no influence of intervention status.

**Table 2:** Association between latrine ownership, sanitation insecurity, individual and cluster level covariates and well-being, anxiety, depression, and distress scores (WHO5) in rural Orissa, India Full models. (Participants=1347, Communities=60).

	**Fixed effects**															
	*Parameter estimate, standard error, confidence interval, p-value*															
**Parameter**	**Well-being**				**Anxiety**				**Depression**				**Distress**			
**Intercept, γ**_**00**_	4.6	1.77	(8.1, 1.1)	0.012*	1.88	0.14	(2.2, 1.6)	<0.001*	2.15	0.15	(2.4, 1.9)	<0.001*	2.04	0.13	(2.3, 1.8)	<0.001*
*Level 1 (individual)*																
**Ownership of a functional latrine, γ**_**10**_	3.4	0.76	(4.9, 1.9)	<0.001*	−0.05	0.06	(0.1, −0.2)	0.430	−0.04	0.06	(0.1, −0.2)	0.554	−0.04	0.06	(0.1, −0.2)	0.452
**Sanitation insecurity**																
1: Potential Harms, γ_20_	−1.3	0.47	(−0.4, −2.2)	0.007*	0.01	0.04	(0.1, −0.1)	0.715	0.13	0.04	(0.2, 0.1)	<0.001*	0.08	0.03	(0.2, 0.0)	0.020*
2: Social expectations & repercussions, γ_30_	0.8	0.72	(2.2, −0.6)	0.276	−0.23	0.06	(−0.1, −0.3)	<0.001*	−0.25	0.06	(−0.1, −0.4)	<0.001*	−0.24	0.05	(−0.1, −0.3)	<0.001*
3: Physical exertion or strain, γ_40_	−3.1	0.86	(−1.4, −4.7)	<0.001*	0.55	0.07	(0.7, 0.4)	<0.001*	0.62	0.07	(0.8, 0.5)	<0.001*	0.59	0.06	(0.7, 0.5)	<0.001*
4: Night Concerns, γ_50_	−0.6	0.26	(−0.1, −1.1)	0.024*	0.22	0.02	(0.3, 0.2)	<0.001*	0.12	0.02	(0.2, 0.1)	<0.001*	0.17	0.02	(0.2, 0.1)	<0.001*
5: Dependent support, γ_60_	−0.4	0.61	(0.7, −1.6)	0.458	0.00	0.05	(0.1, −0.1)	0.975	−0.01	0.05	(0.1, −0.1)	0.812	−0.01	0.04	(0.1, −0.1)	0.885
6: Physical agility, γ_70_	−1.4	0.34	(−0.7, −2.1)	<0.001*	0.00	0.03	(0.1, −0.1)	0.900	0.00	0.03	(0.1, −0.1)	0.868	0.00	0.02	(0.1, 0.0)	0.862
7: Defecation place, γ_80_	1.4	0.50	(2.4, 0.4)	0.005*	0.02	0.04	(0.1, −0.1)	0.599	0.04	0.04	(0.1, 0.0)	0.356	0.03	0.04	(0.1, 0.0)	0.404
**Life stage (Stage 1: Unmarried as referent)**																
Stage 2: Recently Married, γ_90_	−1.3	0.63	(0.0, −2.5)	0.047*	0.16	0.05	(0.3, 0.1)	0.002*	0.15	0.05	(0.2, 0.0)	0.005*	0.15	0.05	(0.2, 0.1)	0.001*
Stage 3: Married over 3 years, γ_100_	−2.7	0.57	(−1.6, −3.8)	<0.001*	0.13	0.05	(0.2, 0.0)	0.003*	0.15	0.05	(0.2, 0.1)	0.001*	0.14	0.04	(0.2, 0.1)	<0.001*
Stage 4: Over 49 years old, γ_110_	−4.3	0.72	(−2.9, −5.7)	<0.001*	0.29	0.06	(0.4, 0.2)	<0.001*	0.39	0.06	(0.5, 0.3)	<0.001*	0.34	0.05	(0.5, 0.2)	<0.001*
**Water access within dwelling/compound, γ**_**120**_	0.5	0.55	(1.6, −0.6)	0.358	0.06	0.04	(0.1, 0.0)	0.202	−0.05	0.05	(0.0, −0.1)	0.265	0.00	0.04	(0.1, −0.1)	0.946
**Bathing area within dwelling/compound, γ**_**130**_	1.8	0.73	(3.2, 0.3)	0.016*	−0.09	0.06	(0.0, −0.2)	0.114	−0.03	0.06	(0.1, −0.1)	0.606	−0.06	0.05	(0.1, −0.2)	0.267
**Possession of 'BPL' card, γ**_**140**_	0.4	0.46	(1.3, −0.5)	0.403	0.04	0.04	(0.1, 0.0)	0.341	0.05	0.04	(0.1, 0.0)	0.180	0.04	0.03	(0.1, 0.0)	0.192
**No current illness, γ**_**150**_	2.4	0.58	(3.5, 1.2)	<0.001*	−0.21	0.05	(−0.1, −0.3)	<0.001*	−0.27	0.05	(−0.2, −0.4)	<0.001*	−0.24	0.04	(−0.2, −0.3)	<0.001*
**Social support, γ**_**160**_	1.6	0.24	(2.1, 1.1)	<0.001*	−0.03	0.02	(0.0, −0.1)	0.130	−0.08	0.02	(0.0, −0.1)	<0.001*	−0.06	0.02	(0.0, −0.1)	<0.001*
*Level 2 (community)*																
**Intervention status, γ**_**01**_	0.5	0.59	(1.6, −0.7)	0.411	0.09	0.05	(0.2, 0.0)	0.070	0.05	0.05	(0.2, −0.1)	0.357	0.07	0.07	(0.2, −0.1)	0.162
	
	**Random parameters**															
	*Variance Component, Standard Deviation, p-value*															
Intercept, u0	1.6	1.3	<0.001*		0.01	0.1	<0.001*			0.02	0.1	<0.001*		0.01	0.1	<0.001*
Level-1, *r*	39.4	6.3			0.25	0.5				0.27	0.5			0.21	0.5	
	**Additional model components**															
ICC (from unconditional model)		0.1				0.07					0.08				0.1	
Deviance		8809.3				2013.4					2086.1				1756.0	
# Estimated Parameters		20.0				20.0					20.0				20.0	
Variance Reduction,τ00		0.0				0.1					0.4				0.1	
Variance Reduction, ᵟ^2^		0.0				0.0					0.0				0.0	
AIC		−8769.3				−1973.4					−2046.06				−1716.0	
BIC		−8665.2				−1869.3					−1941.95				−1611.9	

* Significant at p < 0.05.

#### 3.3.2 Anxiety

In the final model, there was no association between functional latrine access and anxiety scores (β = -0.05, P = 0.43; Table 2), despite an association with reduced anxiety scores in the bivariate model (Model 2; β = -0.15, P < 0.001). Two sanitation insecurity domains, ‘Physical exertion or strain’ and ‘Night concerns’ were positively associated with anxiety scores. One domain, ‘Social expectations and resultant repercussions’, had a negative association. There was an association between life stage and anxiety, with increasing effect as life stage progressed. There was no association between intervention status and anxiety.

#### 3.3.3 Depression

In the final model, there was no association between functional latrine access and depression scores (β = -0.04, P = 0.554; Table 2), though there was a negative (reduction in depression) association in the bivariate model (Model 2; β = -0.17, P < 0.001). Three sanitation insecurity domains were associated with higher depression scores: ‘Potential harms’, ‘ Physical exertion or strain’, and ‘Night concerns’. Similar to the final anxiety model, ‘Social expectations and resultant repercussions’ had a negative effect on depression scores (β = -0.25, P < 0.001). There was a significant effect of life stage on depression scores and no association between intervention status and depression.

#### 3.3.4 Non-specific emotional distress

In the final model, there was no association between functional latrine access and distress scores (β = -0.04, P = 0.452; Table 2), despite a negative association in the bivariate model (Model 2; β = -0.16, P < 0.001). Three sanitation insecurity domains were significantly associated with higher depression scores: ‘Potential harms’, ‘Physical exertion or strain’, and ‘Night concerns’. ‘Social expectations and resultant repercussions’ had a negative association with distress scores (β = -0.24, P < 0.001). There was a significant association of life stage, and no association between intervention status and distress.

## 4 Discussion

We investigated associations between functional household-latrine access and sanitation experience, using the Sanitation Insecurity measure, with mental well-being, and symptoms of anxiety, depression, and distress among women in rural India. While access to a functional household latrine was associated with higher mental well-being scores, access was not associated with anxiety, depression or distress symptoms scores once sanitation insecurity was considered. Women’s sanitation insecurity domains were associated with all four outcomes, with most associated with poorer scores for each, independent of access to a functional household latrine.

These findings suggest that women in rural Orissa, India may suffer assaults to their mental well-being and have high levels of anxiety, depression, and distress when urinating and defecating as a result of experiencing sanitation insecurity, even if they have a facility. Our results align with qualitative findings, which report that women may experience psychosocial impacts related to sanitation behaviors despite facility access (Bisung and Elliott, 2016a, Bisung and Elliott, 2017; Hirve et al., 2015; Sahoo et al., 2015). This research moves beyond these studies, identifying quantitative associations with multiple mental health outcomes, and supports the argument that sanitation-related programs should include intervention components beyond technology to comprehensively impact women’s health (Caruso, Clasen, Hadley, et al., 2017).

These findings have implications for both mental health and sanitation research and programs. This study provides data on four mental health outcomes from a random sample of women at distinct life stages in rural India demonstrating that scores for well-being and symptoms of anxiety, depression, and distress are modest to poor overall and are particularly concerning for older women. Current estimates indicate that the global burden of mental illness results in 32% of years lived with a disability—ranking first in this category—and 13% of disability adjusted life years (Vigo, Thornicroft, & Atun, 2016). The burden of mental health has increased substantially in the past several decades, resulting in social and economic consequences for those afflicted and their families and caregivers (Patel, Chisholm, et al., 2016; Patel, Saxena, Frankish, & Boyce, 2016). There is a particular need to address mental health in low and middle income countries, where needs are high and services are limited. In India, less than 1% of the national budget is spent on mental health, and those living in rural areas have the least access to care (Patel, Xiao, et al., 2016). The present study does not identify the extent that sanitation experiences and access contribute to common mental disorders, but results indicate that women’s experiences urinating and defecating may contribute to mental health states. Interventions designed to ameliorate the sanitation challenges women face could influence mental health outcomes, justifying evaluations of the mental health impacts of sanitation programs. In this regard, it is therefore not surprising that the previous intervention status of the villages had no impact on the outcomes of interest. The intervention was focused on the construction of latrines and was not specifically designed with the specific challenges and needs of women in mind.

The contextually-grounded sanitation insecurity measure enables the identification of specific domains of influence on well-being and symptoms of anxiety, depression, and distress. Two domains of sanitation insecurity, specifically ‘Physical exertion or strain’ and ‘Night concerns’, were significantly associated with poorer scores for all four outcomes, regardless of whether or not women had access to a functional household latrine. The effect of the ‘Physical exertion or strain’ domain highlights the importance of physical challenges associated with managing urination and defecation needs, such as accessing water, or washing the self or clothes afterwards. Qualitative research has reported women’s physical urination and defecation challenges. Sahoo et al. (2015) found water fetching, post-defecation cleaning, and bathing were necessary urination and defecation behaviors that induced stress; Hulland et al. (2015) found fetching water for sanitation-related needs to be among the most stressful activities for women; and Routray et al. (2015) reported that participants would practice open defecation despite owning a latrine because water fetching for anal-cleansing, flushing, and post-defecation bathing and clothes washing was unnecessary if they defecated in the open and accessed nearby water bodies for these activities afterwards. Sanitation programs that do not address the physical exertion women may endure when urinating or defecating, therefore, may not only fail to enable and sustain use, but may also miss an opportunity to improve well-being and reduce anxiety, depression and distress associated with the physical demands of practicing these behaviors.

Women’s fears associated with urination and defecation at night were associated with all outcomes investigated. While women in India often chose to defecate in the cover of darkness to hide themselves and their activities (Caruso, Clasen, Hadley, et al., 2017; Routray et al., 2015), darkness causes fear. To manage fear, women have reported seeking company, suppressing needs at night, or avoiding food and water in the evenings (Caruso et al., 2017, Hulland et al., 2015, Sahoo et al., 2015). Pregnant women feared not only for themselves, but for their unborn children, reporting that a fright in the dark could harm their baby and potentially result in miscarriage (Caruso, Clasen, Hadley, et al., 2017; Sahoo et al., 2015). Latrines in the study villages were all located outside, several meters from the house. Women with no lights inside latrines said they would typically defecate outside a latrine at night due to fear; others reported that having lights in their yards, whether they had a latrine or not, would make night defecation and urination less frightening (Caruso, Clasen, Hadley, et al., 2017). Sanitation programs that address ‘Night concerns’, potentially with low-cost lights, may not only impact mental health, but may also reduce fecal pathogens in the environment by facilitating latrine use.

Higher scores in the domain ‘Potential harms’, which focuses on women’s concerns about harm from people, animals, disease, and dirty conditions, were significantly associated with lower well-being scores and higher depression and distress scores. Studies increasingly have documented women’s experiences and fear of assault related to sanitation behaviors (Caruso, Clasen, Hadley, et al., 2017; Jadhav et al., 2016; Kulkarni et al., 2017; Routray et al., 2015; Sahoo et al., 2015; Winter & Barchi, 2016). Our findings demonstrate that the fear of violence and harm, from men or other sources, has negative associations with mental health outcomes and supports the need to enhance women’s sanitation-related safety and decision making (O’Reilly, 2016; Routray et al., 2017).

The domain ‘Physical agility’, focusing on women’s experiences and concerns falling and squatting when urinating and defecating, had a significant impact on well-being. Pregnant women, older women, and disabled populations have all voiced these concerns (Caruso, Clasen, Hadley, et al., 2017; FANSA & WSSCC, 2015), justifying enhancements to stability providing sanitation structures.

Two domains of sanitation insecurity were associated with the outcomes in unanticipated directions. Higher scores in the domain ‘Defecation place’ were positively associated with well-being scores, potentially because two of the items only ascertained if the experience happened, and were not designed to determine if the experiences were perceived to be negative. Specifically, women were asked how frequently defecation ‘took a long time’ or required them to go far. From qualitative research, many women report defecation to be enjoyable specifically because it provides the opportunity to spend time away from the house and to ‘roam’ with friends (Caruso, Clasen, Hadley, et al., 2017; Coffey et al., 2014; Routray et al., 2015). In future applications of the tool, we recommend adapting the language of these two items to ascertain if the behaviors are concerning.

Higher values on the domain ‘Social expectations and resultant repercussions’ were significantly associated with lower anxiety, depression, and distress scores. Items in this domain focused on suppression because of social constraints, such as obligations or lack of privacy. This domain may not have performed as hypothesized because the items are related to what it means for women in these communities to be, as Joshi et al. (2011) note, ‘a good woman’. Women are expected to tend to needs only at specific times, when people are not around, or when they have no work that takes priority (Khanna & Das, 2015). Answering positively to these questions, therefore, may be a demonstration that they are sacrificing or performing their roles as expected and thus do not suffer anxiety, depression or distress from not meeting expectations. Follow-up research on this domain is warranted.

### 4.1 Strengths and limitations

This study fills a research gap by quantitatively assessing both sanitation access and experience on a range of mental health outcomes with a population-based sample of women representing four unique life stages. Still, limits to causal inference due to the cross sectional design remain.

We did not collect data from a large sample of pregnant women, or engage women younger than 18 or who were too infirm to participate, thus missing these perspectives. Excluding men prevents understanding of how they differ from women. Our focus on women was justified given the qualitative research that has explicitly described their sanitation experiences to be stress inducing. Further research should incorporate broader populations.

This research has enabled assessment of sanitation beyond access, but it does not capture all sanitation-related experiences women may have. Managing menstruation is challenging for women in rural India (Caruso et al., 2017, Hulland et al., 2015, Sahoo et al., 2015) and the sanitation insecurity measure does not address menstruation. Future research should evaluate menstruation experiences in a similar manner.

### 4.2 Conclusion

Among rural women over age 18 in Puri district, Odisha, India, women’s sanitation experiences have mixed associations with well-being, anxiety, depression and distress, despite access to a functional household latrine. Given similarities in physical and social environments across the state, findings are likely to be similar for women throughout Odisha. Future research should continue to explore sanitation experiences to better understand these associations and to assess mental health outcomes associated with sanitation to determine if similar conclusions are reached within this population and others. If so, sanitation initiatives that aim to ameliorate negative experiences of sanitation, beyond the access to facilities, to improve overall health are warranted.
