# Supplementary material for: The association between women's sanitation experiences and mental health: A cross-sectional study in Rural, Odisha India
Source: SSM Popul Health. 2018 Jun 20;5:257–66. doi: 10.1016/j.ssmph.2018.06.005 (PMC6077264; doi:10.1016/j.ssmph.2018.06.005)
Supplement: Supplementary file 1 — Supplementary material [file mmc1.docx]

**Supplemental Material:**

**The association between women’s sanitation experiences and mental health:**

**A cross-sectional study in Rural, Odisha India**

Bethany A Caruso, Hannah LF Cooper, Regine Haardörfer, Kathryn M Yount, Parimita Routray, Belen Torondel, Thomas Clasen

*Social Science & Medicine – Population Health*

**S1 Text: Data Collection and training**

**Data collection Training**

Over the course of four days, we trained a team of nine female data collectors and two supervisors to collect data, and pilot the survey in two communities not selected for inclusion. Training involved (1) a careful review of the survey instrument to make sure all survey questions and methods of recording answers were clear; (2) guidance on survey administration procedures (i.e. assuring that surveys were conducted in a private location to assure confidentiality); and (3) instruction for reading informed consent and seeking ethical approval prior to survey administration. For quality control, we trained supervisors to review each survey as it was completed to be sure all questions were complete and answered appropriately.

**S2 Text: STROBE Checklist**

STROBE Statement—Checklist of items that should be included in reports of ***cross-sectional studies***

**Completed for*:*** The association between women’s sanitation experiences and mental health: A cross-sectional study in Rural, Odisha India

|  | Item No | Recommendation |
| --- | --- | --- |
| **Title and abstract** | 1 | (*a*) Indicate the study’s design with a commonly used term in the title or the abstract  ***Authors:*** The study cross-sectional design of the study is noted explicitly in both the title and in the abstract.  *Title:* The association between women’s sanitation experiences and mental health: A cross-sectional study in Rural, Odisha India |
|  |  | (*b*) Provide in the abstract an informative and balanced summary of what was done and what was found  ***Authors:*** The abstract provides a succinct background, methods that clearly articulate the study design and measures used, the analyses performed, key findings, and conclusions drawn. |
| Introduction | | |
| Background/rationale | 2 | Explain the scientific background and rationale for the investigation being reported  ***Authors:*** We provide the scientific background and rationale for the study in the introduction. |
| Objectives | 3 | State specific objectives, including any prespecified hypotheses  ***Authors:*** The specific objectives and associated hypotheses of the study are noted:  ‘This paper aims to determine quantitatively if sanitation is associated with mental well-being, and symptoms of anxiety, depression, and distress among women in rural Odisha, India. We evaluate these relationships using two sanitation-related exposures: access to a functional household latrine, and “Sanitation Insecurity” (SI), a measure created from the voiced concerns of women to assess the frequency of their negative sanitation experiences. Recognizing that women have varied experiences and needs at different life stages, we incorporate life stage into our model to determine influence.’ |
| Methods | | |
| Study design | 4 | Present key elements of study design early in the paper  ***Authors:*** We provide key elements of the study design in the first paragraph of the methods section:  ‘We conducted a cross-sectional study to evaluate the association between the sanitation exposures and selected mental health outcomes.’ |
| Setting | 5 | Describe the setting, locations, and relevant dates, including periods of recruitment, exposure, follow-up, and data collection  ***Authors:*** We provide descriptions of the setting, dates, and relevant information about the data collection in the first paragraph of the methods:  ‘We conducted a cross-sectional study to evaluate the association between the sanitation exposures and selected mental health outcomes. Data were collected from December 2014-February 2015 in rural communities of Odisha, India, where open defecation is the norm. Our study took place in communities that previously participated in a cluster randomized controlled trial (CRT) designed to assess the impact of a sanitation intervention on diarrhea, soil-transmitted helminth infection, and child malnutrition. The intervention did not result in improvements in any of the health outcomes.’ |
| Participants | 6 | (*a*) Give the eligibility criteria, and the sources and methods of selection of participants  ***Authors:*** We provide eligibility criteria and sources/methods of participant selection of the methods section:  ‘We used a stratified, multi-stage, cluster sample design. The study sampled two units: communities and women living in these communities. We identified 60 communities from the 100 that were engaged in the aforementioned CRT, 30 former intervention and 30 former control, to determine influence of previous intervention status on outcomes. To be eligible, former intervention communities needed to have latrine coverage greater than 25%, and former control communities needed to have latrine coverage less than 20%. These sanitation cut-points were intended to serve as proxies for moderate and poor coverage. We used data from the final trial data collection round (December 2014) to select former intervention communities, assuming little change in coverage (33). For former control communities, we sought data from a non-government organization (NGO) partner actively working to provide sanitation in these communities. Communities were deemed ineligible if they had participated in any of the qualitative research activities undertaken to inform the current study (Anonymous). Thirty-one communities in each arm met our eligibility criteria; we selected the 30 intervention communities with the greatest coverage and the 30 control communities with the least coverage.  We sought to recruit 24 women living in each community, with variation in the sample by life stage given reported differences in sanitation experiences. To create a sampling frame, we conducted a census in each selected community to identify women over 18 years of age in each of four life stages: (1) unmarried, (2) married three years or less, (3) married over three years and age 49 or younger, and (4) over 49 years of age of any marital status. We generated four sampling lists per community, one for each life stage category. Women were eligible to participate if they were randomly selected from one of the lists.’ |
| Variables | 7 | Clearly define all outcomes, exposures, predictors, potential confounders, and effect modifiers. Give diagnostic criteria, if applicable  ***Authors:*** We define outcomes, exposures, and covariates in detail.  A) The four key outcomes (well-being and symptoms of anxiety, depression, and distress) are described. Specifically, we describe:  (1) The World Health Organization Well being Index (WHO-5) to measure subjective mental well being;  (2) The Hopkins Symptoms Checklist (HSCL) to assess symptoms of anxiety, depression, and non-specific emotional distress.  B) The primary exposures, access to a functional latrine in the household compound and *Sanitation Insecurity,* are described. Specifically, we describe:  (1) Access to a functional latrine.  (2) Sanitation Insecurity.  C) The covariates are then described. |
| Data sources/ measurement | 8* | For each variable of interest, give sources of data and details of methods of assessment (measurement). Describe comparability of assessment methods if there is more than one group  ***Authors:*** See ‘variables’ and ‘participants’ sections above for related information. Additional related information on data collection/source of data is provided in ‘Data collection and management’ section of methods:  ‘Enumerators sought to survey six women per life stage category in each community, as available. They skipped eligible participants if another woman in the household had already participated. All surveys were conducted in Oriya, the local language, and responses were recorded using pen and paper by eleven trained female enumerators. Data was double entered and all inconsistencies were checked against surveys and corrected (See S1 Text for additional data collection information).’ |
| Bias | 9 | Describe any efforts to address potential sources of bias  ***Authors:*** We wanted to have the same participants for each outcome and thus excluded participants that were missing predictor or outcome data. Those excluded represent approximately the same percentage from former intervention and former control communities, hence we do not see this as a potential source of bias. See below and flow diagram (figure 1):  ‘As we wanted to include the same participants in each outcome modeled, we excluded 62 participants that had missing predictor or outcome data, representing 4% of the overall sample.’ |
| Study size | 10 | Explain how the study size was arrived at  ***Authors:*** We describe sample size:  ‘A simulation study to informed sample size. This simulation demonstrated power to detect 20% direct and cross-level interaction effects using multilevel (hierarchical) modeling for a continuous level-2 predictor to be greater than 96% for 60 clusters of 20 participants(35). Power was sufficient for both continuous and dichotomous predictors in a base sample size of 1200. We aimed to sample 1440 individuals (24 per community) anticipating 20% non-response due to 1) incomplete surveys or 2) sampling error (i.e. ineligible or misclassified participants).’ |
| Quantitative variables | 11 | Explain how quantitative variables were handled in the analyses. If applicable, describe which groupings were chosen and why  ***Authors:*** All quantitative variables, including scoring for analysis, are described in the ‘Measurement’ section, as noted above.  The specifics of how each outcome was modelled are described in the analysis section:  ‘For each outcome, we estimated five successive hierarchical linear models using maximum likelihood estimation to model clustering of individual women (Level 1, L1) within communities (Level 2, L2). In model 1, we estimated an unconditional model to determine the intraclass correlation coefficient (ICC), the proportion of variance that can be explained by the communities (clusters). In model 2, we ran a multilevel bivariate model that regressed the outcomes on latrine ownership. In model 3, we created a model with latrine ownership and sanitation insecurity to determine if sanitation insecurity was associated with the outcomes, accounting for latrine ownership. In model 4, we added all individual-level covariates. In model 5, we added intervention status, the cluster (community) level covariate, to determine influence of previous trial status.  For each outcome, we calculated the proportional reduction in variance and the proportional reduction in prediction error for each successive model, comparing each model to the more parsimonious model created prior.’ |
| Statistical methods | 12 | (*a*) Describe all statistical methods, including those used to control for confounding  ***Authors:*** The statistical methods are described in the analysis section (also noted above). |
|  |  | (*b*) Describe any methods used to examine subgroups and interactions  ***Authors:*** No interactions were considered. No specific sub-group analyses were performed. Life stage was considered a covariate. |
|  |  | (*c*) Explain how missing data were addressed  ***Authors:*** Participants with missing data were excluded from analysis; this is described in the text. This information is also captured in a flow diagram (Figure 1).  ‘As we wanted to include the same participants in each outcome modeled, we excluded 62 participants that had missing predictor or outcome data, representing 4% of the overall sample.’ |
|  |  | (*d*) If applicable, describe analytical methods taking account of sampling strategy  ***Authors:*** Our full model accounts for life stage category at the individual level and previous community intervention status at the cluster level. See analysis section (pasted above in ‘quantitative variables’. |
|  |  | (*e*) Describe any sensitivity analyses  ***Authors:*** No sensitivity analyses were performed. |
| Results | | |
| Participants | 13* | (a) Report numbers of individuals at each stage of study—eg numbers potentially eligible, examined for eligibility, confirmed eligible, included in the study, completing follow-up, and analysed |
|  |  | (b) Give reasons for non-participation at each stage |
|  |  | (c) Consider use of a flow diagram  ***Authors:*** For (a)-(c): Information is captured in the text as well as the accompanying flow diagram (Figure 1):  ‘In the 60 communities engaged, we approached 2,968 women resulting in 1437 women surveyed (See Fig 1 for a flow diagram indicating reasons for non-participation). Of those, 90 were excluded from analysis because: missing outcome or predictor data (62), participation of another household member (20), or they were too young to have participated (8). The final analytic sample consisted of 1347 participants, including 328 unmarried women (25%), 301 recently married women (22%), 376 women married over three years (28%), and 342 women over age 49 (25%).  ’ |
| Descriptive data | 14* | (a) Give characteristics of study participants (eg demographic, clinical, social) and information on exposures and potential confounders |
|  |  | (b) Indicate number of participants with missing data for each variable of interest  ***Authors:*** For (a) & (b): Characteristics of study participants are provided in lines 250-266 in the text, with additional supporting information in Table 1 and in Supplemental Table 1. The number of participants with missing data for variables reported is indicated in the table footnotes.  ‘The final analytic sample consisted of 1347 participants, including 328 unmarried women (25%), 301 recently married women (22%), 376 women married over three years (28%), and 342 women over age 49 (25%).  Almost all women were Hindu (99%); 45% belonged to the general caste, meaning they did not receive caste-based government support; 66% had a BPL card; 80% indicated they were not suffering from a current illness; 30% reported access to a primary water source within the household dwelling or compound, and 15% reported access to a bathing room (Table 1).  Thirty-six percent reported access to a functional household latrine. Access to sanitation, water and bathing areas varied by life stage, with recently married women having the greatest access of these facilities and unmarried women having the least access. Mean scores for all seven sanitation insecurity domains were low overall, ranging from 0.1 (physical exertion /strain) to 1.2 (Night concerns) (Range=0 (never) to 3 (always)). For each of the domains, scores were progressively lower along life stage categories and among women who had access to a latrine compared to women who did not (S1 Table).’ |
| Outcome data | 15* | Report numbers of outcome events or summary measures  ***Authors:*** See Figure 2 and Tables 1 and 2. |
| Main results | 16 | (*a*) Give unadjusted estimates and, if applicable, confounder-adjusted estimates and their precision (eg, 95% confidence interval). Make clear which confounders were adjusted for and why they were included  ***Authors:*** The main results only show adjusted results (Table 2). Supplemental tables 2-6 have unadjusted results for each of the 4 outcomes of interest. |
|  |  | (*b*) Report category boundaries when continuous variables were categorized  ***Authors:*** Definitions for ‘life stage’, which include consideration of age (a continuous variable), are provided in the methods (as explained above) in lines 108-114. |
|  |  | (*c*) If relevant, consider translating estimates of relative risk into absolute risk for a meaningful time period  ***Authors:*** Not applicable. |
| Other analyses | 17 | Report other analyses done—eg analyses of subgroups and interactions, and sensitivity analyses  ***Authors:*** Not applicable. |
| Discussion | | |
| Key results | 18 | Summarise key results with reference to study objectives  ***Authors:*** We summarise key results with reference to study objectives as follows:  ‘We investigated associations between access to a functional household-latrine and sanitation experience, using the Sanitation Insecurity measure, with mental well-being, and symptoms of anxiety, depression, and distress among women in rural India. While we found that access to a functional household latrine was associated with higher mental well-being scores, access was not associated with anxiety, depression or distress symptoms scores once sanitation insecurity was considered. Women’s sanitation insecurity domains were associated with all four outcomes, with most associated with poorer scores for each, independent of access to a functional household latrine.  These findings suggest that women in rural Orissa, India may suffer assaults to their mental well-being and have high levels of anxiety, depression and distress when urinating and defecating as a result of experiencing sanitation insecurity, even if they have an available facility.’ |
| Limitations | 19 | Discuss limitations of the study, taking into account sources of potential bias or imprecision. Discuss both direction and magnitude of any potential bias  ***Authors:*** Limitations are discussed as follows:  ‘This study fills a notable research gap by quantitatively assessing both sanitation access and sanitation experience on a range of mental health outcomes with a population-based sample of women representing four unique life stages. Still, there remain limits to causal inference due to the cross sectional design. Application of the sanitation insecurity measure in a trial assessing mental health outcomes would enable determination of causality.  There are people in these rural Odisha communities about whom we were not able to learn. We did collect data from a large sample of pregnant women, nor did we try to engage girls younger than or women who were too infirm to participate, missing these perspectives. We also did not collect information from men, preventing understanding of how they differ from women. Our focus on women was justified given the qualitative research that has explicitly described their sanitation experiences to be stress inducing. Further research should incorporate broader populations to understand mental health outcomes associated with sanitation access and Sanitation Insecurity.  This research has enabled assessment of sanitation beyond access, but it does not capture all sanitation-related experiences women may have. Managing menstruation is challenging for women in rural India as well(20, 24, 27) and the sanitation insecurity measure did not capture menstruation concerns or experiences. Future research should focus on evaluating menstruation experiences in a similar manner.’ |
| Interpretation | 20 | Give a cautious overall interpretation of results considering objectives, limitations, multiplicity of analyses, results from similar studies, and other relevant evidence  ***Authors:*** We indicate the below:  ‘Among rural women over age 18 in Puri district, Odisha, India, women’s sanitation experiences have mixed associations with well-being, anxiety, depression and distress, despite access to a functional household latrine. Given similarities in physical and social environments across the state, findings are likely to be similar for women throughout Odisha. Future research should continue to explore sanitation experiences to better understand these associations and to assess mental health outcomes associated with sanitation to determine if similar conclusions are reached within this population and among others. If other studies reach conclusions similar to those reached here, sanitation initiatives could consider how to ameliorate negative experiences of sanitation, thinking dynamically beyond the access to facilities, in order to improve overall health.’ |
| Generalisability | 21 | Discuss the generalisability (external validity) of the study results  ***Authors:*** We indicate the below:  ‘Among rural women over age 18 in Puri district, Odisha, India, women’s sanitation experiences have mixed associations with well-being, anxiety, depression and distress, despite access to a functional household latrine. Given similarities in physical and social environments across the state, findings are likely to be similar for women throughout Odisha. Future research should continue to explore sanitation experiences to better understand these associations and to assess mental health outcomes associated with sanitation to determine if similar conclusions are reached within this population and among others. If other studies reach conclusions similar to those reached here, sanitation initiatives could consider how to ameliorate negative experiences of sanitation, thinking dynamically beyond the access to facilities, in order to improve overall health.’ |
| Other information | | |
| Funding | 22 | Give the source of funding and the role of the funders for the present study and, if applicable, for the original study on which the present article is based  ***Authors:*** The study was funded by The Bill and Melinda Gates Foundation. BAC was funded in part by the NIH/NIGMS Institutional Research and Academic Career Development Award (IRACDA), 5K12-GM000680-18. The funders played no role in the analysis or interpretation of data presented in this study. |

| **S1a Table: Sanitation Insecurity Scores, overall and by life stage with latrine status in Rural Orissa, India (N=1347)** | | | | | | | | | | | | | | | | | | |  |  |  |
| --- | --- | --- | --- | --- | --- | --- | --- | --- | --- | --- | --- | --- | --- | --- | --- | --- | --- | --- | --- | --- | --- |
|  |  |  |  |  |  |  |  |  |  |  |  |  |  |  |  |  |  |  |  |  |  |
|  | **All** | | | | | **1. Unmarried (UM)** | | | | | | | **2. Recently Married (RM)** | | | | | | | |  |
|  | **No Latrine** | | | **Latrine** | | | **No Latrine** | | | **Latrine** | | | | **No Latrine** | | | **Latrine** | | | | |
| **Sanitation Insecurity** | **n=864** | | | **n=483** | | | **n=236** | | | **n=92** | | | | **n=158** | | | **n=143** | | | | |
| 1: Potential Harms (Range: 0-3) | 0.96 | (0.8) | 0.46 | | (0.6)* | 1.13 | | (0.8) | 0.63 | | (0.7)* | 1.23 | | | (0.7) | 0.48 | | (0.6)* | |  |  |
| 2: Social Expectations & Repercussions (No Latrine Range: 0-2.2; Latrine Range: 0-1.8) | 0.52 | (0.5) | 0.27 | | (0.3)* | 0.59 | | (0.5) | 0.36 | | (0.4)* | 0.70 | | | (0.5) | 0.29 | | (0.4)* | |  |  |
| 3: Physical Exertion / Strain (No Latrine Range: 0-2.7; Latrine Range: 0-1.8) | 0.13 | (0.3) | 0.08 | | (0.2)* | 0.15 | | (0.4) | 0.11 | | (0.3) | 0.19 | | | (0.4) | 0.07 | | (0.2)* | |  |  |
| 4: Night Concerns (Range: 0-3) | 1.39 | (1.1) | 0.80 | | (1.0)* | 1.72 | | (1.0) | 1.16 | | (1.1)* | 1.96 | | | (1.0) | 1.02 | | (1.0)* | |  |  |
| 5: Social Support (Range: 0-3) | 0.16 | (0.5) | 0.14 | | (0.4) | 0.04 | | (0.2) | 0.02 | | (0.2) | 0.51 | | | (0.7) | 0.34 | | (0.6)* | |  |  |
| 6: Physical Agility (Range: 0-3) | 0.53 | (0.8) | 0.38 | | (0.7)* | 0.24 | | (0.5) | 0.09 | | (0.3)* | 0.41 | | | (0.7) | 0.22 | | (0.5)* | |  |  |
| 7: Defecation Place (No Latrine Range: 0-3; Latrine Range: 0-2.8) | 1.63 | (0.7) | 0.20 | | (0.4)* | 1.67 | | (0.7) | 0.28 | | (0.5)* | 1.71 | | | (0.7) | 0.12 | | (0.3)* | |  |  |
| *P<0.05: Indicates significant difference between those that have a latrine and those that do not. | | | | | | | | | | | | | | | | | | |  |  |  |

| **S1b Table: Sanitation Insecurity Scores, overall and by life stage with latrine status in Rural Orissa, India (N=1347)** | | | | | | | | | | | |
| --- | --- | --- | --- | --- | --- | --- | --- | --- | --- | --- | --- |
|  |  |  |  |  |  |  |  |  |  |  |  |
|  | **3. Married (M)** | | | | **4. Over 49 (OW)** | | | | |  |  |
|  | **No Latrine** | | **Latrine** | | **No Latrine** | | **Latrine** | | | |  |
| **Sanitation Insecurity** | **n=259** | | **n=117** | | **n=211** | | **n=131** | | | |  |
| 1: Potential Harms (Range: 0-3) | 0.94 | (0.8) | 0.42 | (0.5)* | 0.57 | (0.7) | 0.37 | (0.6)* |  |  |  |
| 2: Social Expectations & Repercussions (No Latrine Range: 0-2.2; Latrine Range: 0-1.8) | 0.53 | (0.4) | 0.27 | (0.3)* | 0.31 | (0.3) | 0.19 | (0.2)* |  |  |  |
| 3: Physical Exertion / Strain (No Latrine Range: 0-2.7; Latrine Range: 0-1.8) | 0.11 | (0.3) | 0.06 | (0.1)* | 0.09 | (0.2) | 0.07 | (0.2) |  |  |  |
| 4: Night Concerns (Range: 0-3) | 1.26 | (1.0) | 0.63 | (0.9)* | 0.77 | (1.0) | 0.46 | (0.8)* |  |  |  |
| 5: Social Support (Range: 0-3) | 0.18 | (0.5) | 0.10 | (0.3) | 0.02 | (0.1) | 0.03 | (0.2) |  |  |  |
| 6: Physical Agility (Range: 0-3) | 0.32 | (0.6) | 0.25 | (0.6) | 1.18 | (1.0) | 0.88 | (1.0)* |  |  |  |
| 7: Defecation Place (No Latrine Range: 0-3; Latrine Range: 0-2.8) | 1.67 | (0.7) | 0.16 | (0.4)* | 1.46 | (0.7) | 0.27 | (0.4)* |  |  |  |
| *P<0.05: Indicates significant difference between those that have a latrine and those that do not. | | | | | | | | | | | |

| **S2 Table: Correlations of mental health outcomes** | | | |
| --- | --- | --- | --- |
|  | **1. Well Being** | **2. Anxiety** | **3. Depression** |
| **1. Well Being** |  |  |  |
| **2. Anxiety** | -0.326 |  |  |
| **3. Depression** | -0.495 | 0.671 |  |
| **4. Distress** | -0.461 | 0.889 | 0.936 |

| **S3 Table: Association between latrine ownership, sanitation insecurity, individual and cluster level covariates and well-being scores (WHO5)**  **in rural Orissa, India (Participants=1347, Communities=60)** | | | | | | | | | | | | | | | | | | | | | | | | | | | | | | | | | | | | |  |  |  |
| --- | --- | --- | --- | --- | --- | --- | --- | --- | --- | --- | --- | --- | --- | --- | --- | --- | --- | --- | --- | --- | --- | --- | --- | --- | --- | --- | --- | --- | --- | --- | --- | --- | --- | --- | --- | --- | --- | --- | --- |
|  | **Fixed Effects** | | | | | | | | | | | | | | | | | | | | | | | | | | | | | | | | | | | |  | |  |
|  | *Parameter estimate, standard error, confidence interval, p-value* | | | | | | | | | | | | | | | | | | | | | | | | | | | | | | | | | | | |  | |  |
|  | **Unconditional** | | | | **Ownership of Functional Latrine** | | | | | **Ownership of Functional Latrine and Sanitation Insecurity** | | | | | | | **Ownership of Functional Latrine, Sanitation Insecurity, and Individual Level Covariates** | | | | | | | | | | | **Ownership of Functional Latrine, Sanitation Insecurity, and Individual and Cluster Level Covariates** | | | | | | | | |  |  |  |
| **Parameter** | **Model 1** | | | | **Model 2** | | | | | **Model 3** | | | | | | **Model 4** | | | | | | | | | | | | | **Model 5** | | | | | | | |  | |  |
| **Intercept, γ_00_** | 13.1 | 0.34 | (13.7, 14.4) | <0.001* | 12.1 | 0.37 | (12.9, 11.4) | <0.001* | 12.9 | | 0.73 | | (14.3, 11.5) | | <0.001* | | | 4.9 | 1.74 | | | | (8.3, 1.4) | | | | 0.007* | | | 4.6 | | 1.77 | (8.1, 1.1) | | | 0.012* | |  |  |
| *Level 1 (individual)* | | | |  |  |  |  |  |  | |  | |  | |  | | |  |  | | | |  | | | |  | | |  | |  |  | | |  | |  |  |
| **Ownership of a functional latrine, γ_10_** | | | |  | 2.8 | 0.54 | (3.9, 1.8) | <0.001* | 3.8 | | 0.77 | | (5.3, 2.3) | | <0.001* | | | 3.4 | 0.75 | | | | (4.9, 2.0) | | | | <0.001* | | | 3.4 | | 0.76 | (4.9, 1.9) | | | <0.001* | |  |  |
| **Sanitation Insecurity** |  |  |  |  |  |  |  |  |  | |  | |  | |  | | |  |  | | | |  | | | |  | | |  | |  |  | | |  | |  |  |
| 1: Potential Harms, γ_20_ |  |  |  |  |  |  |  |  | -0.9 | | 0.50 | | (0.1, -1.9) | | 0.078 | | | -1.3 | 0.47 | | | | (-0.4, -2.2) | | | | 0.007* | | | -1.3 | | 0.47 | (-0.4, -2.2) | | | 0.007* | |  |  |
| 2: Social expectations & repercussions, γ_30_ | | | |  |  |  |  |  | 1.2 | | 0.76 | | (2.6, -0.3) | | 0.126 | | | 0.8 | 0.72 | | | | (2.2, -0.6) | | | | 0.276 | | | 0.8 | | 0.72 | (2.2, -0.6) | | | 0.276 | |  |  |
| 3: Physical exertion or strain, γ_40_ | | | |  |  |  |  |  | -1.8 | | 0.92 | | (0.0, -3.6) | | 0.044* | | | -3.0 | 0.86 | | | | (-1.4, -4.7) | | | | <0.001* | | | -3.1 | | 0.86 | (-1.4, -4.7) | | | <0.001* | |  |  |
| 4: Night Concerns, γ_50_ |  |  |  |  |  |  |  |  | -0.1 | | 0.27 | | (0.5, -0.6) | | 0.851 | | | -0.6 | 0.26 | | | | (-0.1, -1.1) | | | | 0.027* | | | -0.6 | | 0.26 | (-0.1, -1.1) | | | 0.024* | |  |  |
| 5: Dependent support, γ_60_ | | |  |  |  |  |  |  | -0.6 | | 0.61 | | (0.6, -1.8) | | 0.365 | | | -0.4 | 0.61 | | | | (0.7, -1.6) | | | | 0.463 | | | -0.4 | | 0.61 | (0.7, -1.6) | | | 0.458 | |  |  |
| 6: Physical agility, γ_70_ |  |  |  |  |  |  |  |  | -3.2 | | 0.30 | | (-2.6, -3.7) | | <0.001* | | | -1.4 | 0.34 | | | | (-0.7, -2.1) | | | | <0.001* | | | -1.4 | | 0.34 | (-0.7, -2.1) | | | <0.001* | |  |  |
| 7: Defecation place, γ_80_ | | | |  |  |  |  |  | 0.9 | | 0.52 | | (1.9, -0.1) | | 0.077 | | | 1.3 | 0.49 | | | | (2.3, 0.4) | | | | 0.007* | | | 1.4 | | 0.50 | (2.4, 0.4) | | | 0.005* | |  |  |
| **Life Stage (Stage 1: Unmarried as referent)** | | | |  |  |  |  |  |  | |  | |  | |  | | |  |  | | | |  | | | |  | | |  | |  |  | | |  | |  |  |
| Stage 2: Recently Married, γ_90_ | | | |  |  |  |  |  |  | |  | |  | |  | | | -1.3 | 0.63 | | | | (0.0, -2.5) | | | | 0.044 | | | -1.3 | | 0.63 | (0.0, -2.5) | | | 0.047* | |  |  |
| Stage 3: Married over 3 years, γ_100_ | | | |  |  |  |  |  |  | |  | |  | |  | | | -2.7 | 0.57 | | | | (-1.6, -3.8) | | | | <0.001* | | | -2.7 | | 0.57 | (-1.6, -3.8) | | | <0.001* | |  |  |
| Stage 4: Over 49 years old, γ_110_ | | | |  |  |  |  |  |  | |  | |  | |  | | | -4.3 | 0.72 | | | | (-2.9, -5.7) | | | | <0.001* | | | -4.3 | | 0.72 | (-2.9, -5.7) | | | <0.001* | |  |  |
| **Water access within dwelling / compound, γ_120_** | | | |  |  |  |  |  |  | |  | |  | |  | | | 0.5 | 0.55 | | | | (1.6, -0.6) | | | | 0.338 | | | 0.5 | | 0.55 | (1.6, -0.6) | | | 0.358 | |  |  |
| **Bathing Area within dwelling / compound, γ_130_** | | | |  |  |  |  |  |  | |  | |  | |  | | | 1.7 | 0.73 | | | | (3.1, 0.3) | | | | 0.020* | | | 1.8 | | 0.73 | (3.2, 0.3) | | | 0.016* | |  |  |
| **Possession of 'BPL' card, γ_140_** | | | |  |  |  |  |  |  | |  | |  | |  | | | 0.4 | 0.46 | | | | (1.3, -0.5) | | | | 0.395 | | | 0.4 | | 0.46 | (1.3, -0.5) | | | 0.403 | |  |  |
| **No current illness, γ_150_** | | | |  |  |  |  |  |  | |  | |  | |  | | | 2.4 | 0.58 | | | | (3.5, 1.2) | | | | <0.001* | | | 2.4 | | 0.58 | (3.5, 1.2) | | | <0.001* | |  |  |
| **Social Support, γ_160_** |  |  |  |  |  |  |  |  |  | |  | |  | |  | | | 1.6 | 0.24 | | | | (2.1, 1.1) | | | | <0.001* | | | 1.6 | | 0.24 | (2.1, 1.1) | | | <0.001* | |  |  |
| *Level 2 (community)* |  |  |  |  |  |  |  |  |  | |  | |  | |  | | |  |  | | | |  | | | |  | | |  | |  |  | | |  | |  |  |
| **Intervention Status, γ_01_** | | |  |  |  |  |  |  |  | |  | |  | |  | | |  |  | | | |  | | | |  | | | 0.5 | | 0.59 | (1.6, -0.7) | | | 0.411 | |  |  |
|  | **Random Parameters** | | | | | | | | | | | | | | | | | | | | | | | | | | | | | | | | | | | | |  |  |
|  | *Variance Component, Standard Deviation, p-value* | | | | | | | | | | | | | | | | | | | | | | | | | | | | | | | | | | | | |  |  |
| Intercept, u0 | 3.0 | 1.7 | <0.001* |  | 2.3 | 1.5 | <0.001* |  | 1.9 | | 1.4 | | <0.001* | |  | | | 1.6 | 1.3 | | | | <0.001* | | | |  | | | 1.6 | | 1.3 | <0.001* | | |  | |  |  |
| Level-1, *r* | 53.6 | 7.3 |  |  | 52.3 | 7.2 |  |  | 45.5 | | 6.7 | |  | |  | | | 39.4 | 6.3 | | | |  | | | |  | | | 39.4 | | 6.3 |  | | |  | |  |  |
|  | **Additional Model Components** | | | | | | | | | | | | | | | | | | | | | | | | | | | | | | | | | | | | |  |  |
| ICC | 0.1 | |  |  |  |  |  |  |  | |  | |  | |  | | |  |  | | | |  | | | |  | | |  | |  |  | | |  | |  |  |
| Deviance | 9232.2 | |  |  | 9193.2 | |  |  | 9004.4 | | | |  | |  | | | 8810.2 | | | | |  | | | |  | | | 8809.3 | | |  | | |  | |  |  |
| # Estimated Parameters | 3.0 | |  |  | 4.0 | |  |  | 11.0 | | | |  | |  | | | 19.0 | | | |  | | | | |  | | | 20.0 | | |  | | |  | |  |  |
| Variance Reduction,τ00 |  |  |  |  | 0.2 | |  |  | 0.2 | | | |  | |  | | | 0.2 | | | |  | | | | |  | | | 0.0 | | |  | | |  | |  |  |
| Variance Reduction, ᵟ^2^ |  |  |  |  | 0.0 | |  |  | 0.1 | | | |  | |  | | | 0.1 | | | |  | | | | |  | | | 0.0 | | |  | | |  | |  |  |
| AIC |  |  |  |  | -9185.2 | |  |  | -8982.4 | | | |  | |  | | | -8772.2 | | |  | | | | |  | | | -8769.3 | | | |  | | |  | |  |  |
| BIC |  |  |  |  | -9164.4 | |  |  | -8925.1 | | | |  | |  | | | -8673.3 | | |  | | | | |  | | | -8665.2 | | | |  | | |  | |  |  |
| *Significant at p <0.05 |  |  |  |  |  |  |  |  | |  | |  | |  | |  | | | |  | | | |  |  | | | |  | |  | | |  |  | | |  | |

| **S4 Table: Association between latrine ownership, sanitation insecurity, individual and cluster level covariates and anxiety scores (HSCL, Q1-10)**  **in rural Orissa, India (Participants=1347, Communities=60)** | | | | | | | | | | | | | | | | | | | | | | | | | | | | | | | | | | | | |  |  |  |
| --- | --- | --- | --- | --- | --- | --- | --- | --- | --- | --- | --- | --- | --- | --- | --- | --- | --- | --- | --- | --- | --- | --- | --- | --- | --- | --- | --- | --- | --- | --- | --- | --- | --- | --- | --- | --- | --- | --- | --- |
|  | **Fixed Effects** | | | | | | | | | | | | | | | | | | | | | | | | | | | | | | | | | | | |  | |  |
|  | *Parameter estimate, standard error, confidence interval, p-value* | | | | | | | | | | | | | | | | | | | | | | | | | | | | | | | | | | | |  | |  |
|  | **Unconditional** | | | | **Ownership of Functional Latrine** | | | | | **Ownership of Functional Latrine and Sanitation Insecurity** | | | | | | | **Ownership of Functional Latrine, Sanitation Insecurity, and Individual Level Covariates** | | | | | | | | | | | **Ownership of Functional Latrine, Sanitation Insecurity, and Individual and Cluster Level Covariates** | | | | | | | | |  |  |  |
| **Parameter** | **Model 1** | | | | **Model 2** | | | | | **Model 3** | | | | | | **Model 4** | | | | | | | | | | | | | **Model 5** | | | | | | | |  | |  |
| **Intercept, γ_00_** | 1.85 | 0.03 | (1.9, 1.8) | <0.001* | 1.90 | 0.03 | (2.0, 1.8) | <0.001* | 1.64 | | 0.06 | | (1.8, 1.5) | | <0.001* | | | 1.93 | 0.14 | | | | (2.2, 1.7) | | | | <0.001* | | | 1.88 | | 0.14 | (2.2, 1.6) | | | <0.001* | |  |  |
| *Level 1 (individual)* | | | |  |  |  |  |  |  | |  | |  | |  | | |  |  | | | |  | | | |  | | |  | |  |  | | |  | |  |  |
| **Ownership of a functional latrine, γ_10_** | | | |  | -0.15 | 0.04 | (-0.1, -0.2) | <0.001* | -0.05 | | 0.06 | | (0.1, -0.2) | | 0.366 | | | -0.04 | 0.06 | | | | (0.1, -0.2) | | | | 0.558 | | | -0.05 | | 0.06 | (0.1, -0.2) | | | 0.430 | |  |  |
| **Sanitation Insecurity** |  |  |  |  |  |  |  |  |  | |  | |  | |  | | |  |  | | | |  | | | |  | | |  | |  |  | | |  | |  |  |
| 1: Potential Harms, γ_20_ |  |  |  |  |  |  |  |  | -0.01 | | 0.04 | | (0.1, -0.1) | | 0.794 | | | 0.01 | 0.04 | | | | (0.1, -0.1) | | | | 0.708 | | | 0.01 | | 0.04 | (0.1, -0.1) | | | 0.715 | |  |  |
| 2: Social expectations & repercussions, γ_30_ | | | |  |  |  |  |  | -0.26 | | 0.06 | | (-0.1, -0.4) | | <0.001* | | | -0.23 | 0.06 | | | | (-0.1, -0.3) | | | | <0.001* | | | -0.23 | | 0.06 | (-0.1, -0.3) | | | <0.001* | |  |  |
| 3: Physical exertion or strain, γ_40_ | | | |  |  |  |  |  | 0.48 | | 0.07 | | (0.6, 0.3) | | <0.001* | | | 0.55 | 0.07 | | | | (0.7, 0.4) | | | | <0.001* | | | 0.55 | | 0.07 | (0.7, 0.4) | | | <0.001* | |  |  |
| 4: Night Concerns, γ_50_ |  |  |  |  |  |  |  |  | 0.20 | | 0.02 | | (0.2, 0.2) | | <0.001* | | | 0.22 | 0.22 | | | | (0.7, -0.2) | | | | <0.001* | | | 0.22 | | 0.02 | (0.3, 0.2) | | | <0.001* | |  |  |
| 5: Dependent support, γ_60_ | | |  |  |  |  |  |  | 0.00 | | 0.05 | | (0.1, -0.1) | | 0.926 | | | 0.00 | 0.05 | | | | (0.1, -0.1) | | | | 0.968 | | | 0.00 | | 0.05 | (0.1, -0.1) | | | 0.975 | |  |  |
| 6: Physical agility, γ_70_ |  |  |  |  |  |  |  |  | 0.12 | | 0.02 | | (0.2, 0.1) | | <0.001* | | | 0.01 | 0.03 | | | | (0.1, 0.0) | | | | 0.846 | | | 0.00 | | 0.03 | (0.1, -0.1) | | | 0.900 | |  |  |
| 7: Defecation place, γ_80_ | | | |  |  |  |  |  | 0.02 | | 0.04 | | (0.1, -0.1) | | 0.705 | | | 0.01 | 0.04 | | | | (0.1, -0.1) | | | | 0.732 | | | 0.02 | | 0.04 | (0.1, -0.1) | | | 0.599 | |  |  |
| **Life Stage (Stage 1: Unmarried as referent)** | | | |  |  |  |  |  |  | |  | |  | |  | | |  |  | | | |  | | | |  | | |  | |  |  | | |  | |  |  |
| Stage 2: Recently Married, γ_90_ | | | |  |  |  |  |  |  | |  | |  | |  | | | 0.16 | 0.05 | | | | (0.3, 0.1) | | | | 0.002* | | | 0.16 | | 0.05 | (0.3, 0.1) | | | 0.002* | |  |  |
| Stage 3: Married over 3 years, γ_100_ | | | |  |  |  |  |  |  | |  | |  | |  | | | 0.13 | 0.05 | | | | (0.2, 0.0) | | | | 0.003* | | | 0.13 | | 0.05 | (0.2, 0.0) | | | 0.003* | |  |  |
| Stage 4: Over 49 years old, γ_110_ | | | |  |  |  |  |  |  | |  | |  | |  | | | 0.28 | 0.06 | | | | (0.4, 0.2) | | | | <0.001* | | | 0.29 | | 0.06 | (0.4, 0.2) | | | <0.001* | |  |  |
| **Water access within dwelling / compound, γ_120_** | | | |  |  |  |  |  |  | |  | |  | |  | | | 0.06 | 0.04 | | | | (0.1, 0.0) | | | | 0.168 | | | 0.06 | | 0.04 | (0.1, 0.0) | | | 0.202 | |  |  |
| **Bathing Area within dwelling / compound, γ_130_** | | | |  |  |  |  |  |  | |  | |  | |  | | | -0.10 | 0.06 | | | | (0.0, -0.2) | | | | 0.080 | | | -0.09 | | 0.06 | (0.0, -0.2) | | | 0.114 | |  |  |
| **Possession of 'BPL' card, γ_140_** | | | |  |  |  |  |  |  | |  | |  | |  | | | 0.04 | 0.04 | | | | (0.1, 0.0) | | | | 0.328 | | | 0.04 | | 0.04 | (0.1, 0.0) | | | 0.341 | |  |  |
| **No current illness, γ_150_** | | | |  |  |  |  |  |  | |  | |  | |  | | | -0.21 | 0.05 | | | | (-0.1, -0.3) | | | | <0.001* | | | -0.21 | | 0.05 | (-0.1, -0.3) | | | <0.001* | |  |  |
| **Social Support, γ_160_** |  |  |  |  |  |  |  |  |  | |  | |  | |  | | | -0.03 | 0.02 | | | | (0.0, -0.1) | | | | 0.123 | | | -0.03 | | 0.02 | (0.0, -0.1) | | | 0.130 | |  |  |
| *Level 2 (community)* |  |  |  |  |  |  |  |  |  | |  | |  | |  | | |  |  | | | |  | | | |  | | |  | |  |  | | |  | |  |  |
| **Intervention Status, γ_01_** | | |  |  |  |  |  |  |  | |  | |  | |  | | |  |  | | | |  | | | |  | | | 0.09 | | 0.05 | (0.2, 0.0) | | | 0.070 | |  |  |
|  | **Random Parameters** | | | | | | | | | | | | | | | | | | | | | | | | | | | | | | | | | | | | |  |  |
|  | *Variance Component, Standard Deviation, p-value* | | | | | | | | | | | | | | | | | | | | | | | | | | | | | | | | | | | | |  |  |
| Intercept, u0 | 0.02 | 0.2 | <0.001* |  | 0.02 | 0.2 | <0.001* |  | 0.02 | | 0.1 | | <0.001* | |  | | | 0.02 | 0.1 | | | | <0.001* | | | |  | | | 0.01 | | 0.1 | <0.001* | | |  | |  |  |
| Level-1, *r* | 0.33 | 0.6 |  |  | 0.33 | 0.6 |  |  | 0.27 | | 0.5 | |  | |  | | | 0.25 | 0.5 | | | |  | | | |  | | | 0.25 | | 0.5 |  | | |  | |  |  |
|  | **Additional Model Components** | | | | | | | | | | | | | | | | | | | | | | | | | | | | | | | | | | | | |  |  |
| ICC | 0.07 | |  |  |  |  |  |  |  | |  | |  | |  | | |  |  | | | |  | | | |  | | |  | |  |  | | |  | |  |  |
| Deviance | 2396.3 | |  |  | 2378.5 | |  |  | 2104.1 | | | |  | |  | | | 2017.7 | | | | |  | | | |  | | | 2013.4 | | |  | | |  | |  |  |
| # Estimated Parameters | 3.0 | |  |  | 4.0 | |  |  | 11.0 | | | |  | |  | | | 19.0 | | | |  | | | | |  | | | 20.0 | | |  | | |  | |  |  |
| Variance Reduction,τ00 |  |  |  |  | -0.1 | |  |  | 0.4 | | | |  | |  | | | -0.1 | | | |  | | | | |  | | | 0.1 | | |  | | |  | |  |  |
| Variance Reduction, ᵟ^2^ |  |  |  |  | 0.2 | |  |  | 0.2 | | | |  | |  | | | 0.1 | | | |  | | | | |  | | | 0.0 | | |  | | |  | |  |  |
| AIC |  |  |  |  | -2370.5 | |  |  | -2082.1 | | | |  | |  | | | -1979.7 | | |  | | | | |  | | | -1973.4 | | | |  | | |  | |  |  |
| BIC |  |  |  |  | -2349.7 | |  |  | -2024.8 | | | |  | |  | | | -1880.8 | | |  | | | | |  | | | -1869.3 | | | |  | | |  | |  |  |
| *Significant at p <0.05 |  |  |  |  |  |  |  |  | |  | |  | |  | |  | | | |  | | | |  |  | | | |  | |  | | |  |  | | |  | |

| **S5 Table: Association between latrine ownership, sanitation insecurity, individual and cluster level covariates and depression scores (HSCL, Q11-23)**  **in rural Orissa, India (Participants=1347, Communities=60)** | | | | | | | | | | | | | | | | | | | | | | | | | | | | | | | | | | | | |  |  |  |
| --- | --- | --- | --- | --- | --- | --- | --- | --- | --- | --- | --- | --- | --- | --- | --- | --- | --- | --- | --- | --- | --- | --- | --- | --- | --- | --- | --- | --- | --- | --- | --- | --- | --- | --- | --- | --- | --- | --- | --- |
|  | **Fixed Effects** | | | | | | | | | | | | | | | | | | | | | | | | | | | | | | | | | | | |  | |  |
|  | *Parameter estimate, standard error, confidence interval, p-value* | | | | | | | | | | | | | | | | | | | | | | | | | | | | | | | | | | | |  | |  |
|  | **Unconditional** | | | | **Ownership of Functional Latrine** | | | | | **Ownership of Functional Latrine and Sanitation Insecurity** | | | | | | | **Ownership of Functional Latrine, Sanitation Insecurity, and Individual Level Covariates** | | | | | | | | | | | **Ownership of Functional Latrine, Sanitation Insecurity, and Individual and Cluster Level Covariates** | | | | | | | | |  |  |  |
| **Parameter** | **Model 1** | | | | **Model 2** | | | | | **Model 3** | | | | | | **Model 4** | | | | | | | | | | | | | **Model 5** | | | | | | | |  | |  |
| **Intercept, γ_00_** | 1.86 | 0.03 | (1.9, 1.8) | <0.001* | 1.91 | 0.04 | (2.0, 1.8) | <0.001* | 1.62 | | 0.06 | | (1.7, 1.5) | | <0.001* | | | 2.18 | 0.14 | | | | (2.5, 1.9) | | | | <0.001 | | | 2.15 | | 0.15 | (2.4, 1.9) | | | <0.001* | |  |  |
| *Level 1 (individual)* | | | |  |  |  |  |  |  | |  | |  | |  | | |  |  | | | |  | | | |  | | |  | |  |  | | |  | |  |  |
| **Ownership of a functional latrine, γ_10_** | | | |  | -0.17 | 0.05 | (-0.1, -0.3) | <0.001* | -0.04 | | 0.06 | | (0.1, -0.2) | | 0.562 | | | -0.03 | 0.06 | | | | (0.1, -0.2) | | | | 0.624 | | | -0.04 | | 0.06 | (0.1, -0.2) | | | 0.554 | |  |  |
| **Sanitation Insecurity** |  |  |  |  |  |  |  |  |  | |  | |  | |  | | |  |  | | | |  | | | |  | | |  | |  |  | | |  | |  |  |
| 1: Potential Harms, γ_20_ |  |  |  |  |  |  |  |  | 0.09 | | 0.04 | | (0.2, 0.0) | | 0.024 | | | 0.13 | 0.04 | | | | (0.2, 0.1) | | | | <0.001* | | | 0.13 | | 0.04 | (0.2, 0.1) | | | <0.001* | |  |  |
| 2: Social expectations & repercussions, γ_30_ | | | |  |  |  |  |  | -0.30 | | 0.06 | | (-0.2, -0.4) | | <0.001* | | | -0.25 | 0.06 | | | | (-0.1, -0.4) | | | | <0.001* | | | -0.25 | | 0.06 | (-0.1, -0.4) | | | <0.001* | |  |  |
| 3: Physical exertion or strain, γ_40_ | | | |  |  |  |  |  | 0.53 | | 0.07 | | (0.7, 0.4) | | <0.001* | | | 0.62 | 0.07 | | | | (0.8, 0.5) | | | | <0.001* | | | 0.62 | | 0.07 | (0.8, 0.5) | | | <0.001* | |  |  |
| 4: Night Concerns, γ_50_ |  |  |  |  |  |  |  |  | 0.08 | | 0.02 | | (0.1, 0.0) | | <0.001* | | | 0.12 | 0.02 | | | | (0.2, 0.1) | | | | <0.001* | | | 0.12 | | 0.02 | (0.2, 0.1) | | | <0.001* | |  |  |
| 5: Dependent support, γ_60_ | | |  |  |  |  |  |  | -0.01 | | 0.05 | | (0.1, -0.1) | | 0.860 | | | -0.01 | 0.05 | | | | (0.1, -0.1) | | | | 0.812 | | | -0.01 | | 0.05 | (0.1, -0.1) | | | 0.812 | |  |  |
| 6: Physical agility, γ_70_ |  |  |  |  |  |  |  |  | 0.18 | | 0.02 | | (0.2, 0.1) | | <0.001* | | | 0.01 | 0.03 | | | | (0.1, 0.0) | | | | 0.843 | | | 0.00 | | 0.03 | (0.1, -0.1) | | | 0.868 | |  |  |
| 7: Defecation place, γ_80_ | | | |  |  |  |  |  | 0.06 | | 0.04 | | (0.1, 0.0) | | 0.166 | | | 0.03 | 0.04 | | | | (0.1, 0.0) | | | | 0.403 | | | 0.04 | | 0.04 | (0.1, 0.0) | | | 0.356 | |  |  |
| **Life Stage (Stage 1: Unmarried as referent)** | | | |  |  |  |  |  |  | |  | |  | |  | | |  |  | | | |  | | | |  | | |  | |  |  | | |  | |  |  |
| Stage 2: Recently Married, γ_90_ | | | |  |  |  |  |  |  | |  | |  | |  | | | 0.14 | 0.05 | | | | (0.2, 0.0) | | | | 0.006* | | | 0.15 | | 0.05 | (0.2, 0.0) | | | 0.005* | |  |  |
| Stage 3: Married over 3 years, γ_100_ | | | |  |  |  |  |  |  | |  | |  | |  | | | 0.15 | 0.05 | | | | (0.2, 0.1) | | | | 0.001* | | | 0.15 | | 0.05 | (0.2, 0.1) | | | 0.001* | |  |  |
| Stage 4: Over 49 years old, γ_110_ | | | |  |  |  |  |  |  | |  | |  | |  | | | 0.39 | 0.06 | | | | (0.5, 0.3) | | | | <0.001* | | | 0.39 | | 0.06 | (0.5, 0.3) | | | <0.001* | |  |  |
| **Water access within dwelling / compound, γ_120_** | | | |  |  |  |  |  |  | |  | |  | |  | | | -0.05 | 0.05 | | | | (0.0, -0.1) | | | | 0.288 | | | -0.05 | | 0.05 | (0.0, -0.1) | | | 0.265 | |  |  |
| **Bathing Area within dwelling / compound, γ_130_** | | | |  |  |  |  |  |  | |  | |  | |  | | | -0.04 | 0.06 | | | | (0.1, -0.2) | | | | 0.552 | | | -0.03 | | 0.06 | (0.1, -0.1) | | | 0.606 | |  |  |
| **Possession of 'BPL' card, γ_140_** | | | |  |  |  |  |  |  | |  | |  | |  | | | 0.05 | 0.04 | | | | (0.1, 0.0) | | | | 0.178 | | | 0.05 | | 0.04 | (0.1, 0.0) | | | 0.180 | |  |  |
| **No current illness, γ_150_** | | | |  |  |  |  |  |  | |  | |  | |  | | | -0.27 | 0.05 | | | | (-0.2, -0.4) | | | | <0.001* | | | -0.27 | | 0.05 | (-0.2, -0.4) | | | <0.001* | |  |  |
| **Social Support, γ_160_** |  |  |  |  |  |  |  |  |  | |  | |  | |  | | | -0.08 | 0.02 | | | | (0.0, -0.1) | | | | <0.001* | | | -0.08 | | 0.02 | (0.0, -0.1) | | | <0.001* | |  |  |
| *Level 2 (community)* |  |  |  |  |  |  |  |  |  | |  | |  | |  | | |  |  | | | |  | | | |  | | |  | |  |  | | |  | |  |  |
| **Intervention Status, γ_01_** | | |  |  |  |  |  |  |  | |  | |  | |  | | |  |  | | | |  | | | |  | | | 0.05 | | 0.05 | (0.2, -0.1) | | | 0.357 | |  |  |
|  | **Random Parameters** | | | | | | | | | | | | | | | | | | | | | | | | | | | | | | | | | | | | |  |  |
|  | *Variance Component, Standard Deviation, p-value* | | | | | | | | | | | | | | | | | | | | | | | | | | | | | | | | | | | | |  |  |
| Intercept, u0 | 0.03 | 0.2 | <0.001* |  | 0.03 | 0.1 | <0.001* |  | 0.02 | | 0.1 | | <0.001* | |  | | | 0.02 | 0.1 | | | | <0.001* | | | |  | | | 0.02 | | 0.1 | <0.001* | | |  | |  |  |
| Level-1, *r* | 0.36 | 0.6 |  |  | 0.36 | 0.6 |  |  | 0.30 | | 0.5 | |  | |  | | | 0.26 | 0.5 | | | |  | | | |  | | | 0.27 | | 0.5 |  | | |  | |  |  |
|  | **Additional Model Components** | | | | | | | | | | | | | | | | | | | | | | | | | | | | | | | | | | | | |  |  |
| ICC | 0.08 | |  |  |  |  |  |  |  | |  | |  | |  | | |  |  | | | |  | | | |  | | |  | |  |  | | |  | |  |  |
| Deviance | 2523.3 | |  |  | 2504.2 | |  |  | 2256.5 | | | |  | |  | | | 2087.2 | | | | |  | | | |  | | | 2086.1 | | |  | | |  | |  |  |
| # Estimated Parameters | 3.0 | |  |  | 4.0 | |  |  | 11.0 | | | |  | |  | | | 19.0 | | | |  | | | | |  | | | 20.0 | | |  | | |  | |  |  |
| Variance Reduction,τ00 |  |  |  |  | 0.1 | |  |  | 0.4 | | | |  | |  | | | 0.1 | | | |  | | | | |  | | | 0.4 | | |  | | |  | |  |  |
| Variance Reduction, ᵟ^2^ |  |  |  |  | 0.0 | |  |  | 0.2 | | | |  | |  | | | 0.1 | | | |  | | | | |  | | | 0.0 | | |  | | |  | |  |  |
| AIC |  | |  |  | -2496.22 | |  |  | -2234.50 | | | |  | |  | | | -2049.2 | | |  | | | | |  | | | -2046.06 | | | |  | | |  | |  |  |
| BIC |  | |  |  | -2475.40 | |  |  | -2177.24 | | | |  | |  | | | -1950.2 | | |  | | | | |  | | | -1941.95 | | | |  | | |  | |  |  |
| *Significant at p <0.05 |  |  |  |  |  |  |  |  | |  | |  | |  | |  | | | |  | | | |  |  | | | |  | |  | | |  |  | | |  | |

| **S6 Table: Association between latrine ownership, sanitation insecurity, individual and cluster level covariates and non-specific emotional distress scores (HSCL, Q1-23) in rural Orissa, India (Participants=1347, Communities=60)** | | | | | | | | | | | | | | | | | | | | | | | | | | | | | | | | | | | | |  |  |  |
| --- | --- | --- | --- | --- | --- | --- | --- | --- | --- | --- | --- | --- | --- | --- | --- | --- | --- | --- | --- | --- | --- | --- | --- | --- | --- | --- | --- | --- | --- | --- | --- | --- | --- | --- | --- | --- | --- | --- | --- |
|  | **Fixed Effects** | | | | | | | | | | | | | | | | | | | | | | | | | | | | | | | | | | | |  | |  |
|  | *Parameter estimate, standard error, confidence interval, p-value* | | | | | | | | | | | | | | | | | | | | | | | | | | | | | | | | | | | |  | |  |
|  | **Unconditional** | | | | **Ownership of Functional Latrine** | | | | | **Ownership of Functional Latrine and Sanitation Insecurity** | | | | | | | **Ownership of Functional Latrine, Sanitation Insecurity, and Individual Level Covariates** | | | | | | | | | | | **Ownership of Functional Latrine, Sanitation Insecurity, and Individual and Cluster Level Covariates** | | | | | | | | |  |  |  |
| **Parameter** | **Model 1** | | | | **Model 2** | | | | | **Model 3** | | | | | | **Model 4** | | | | | | | | | | | | | **Model 5** | | | | | | | |  | |  |
| **Intercept, γ_00_** | 1.85 | 0.03 | (1.9, 1.8) | <0.001* | 1.91 | 0.03 | (2.0, 1.8) | <0.001* | 1.63 | | 0.05 | | (1.7, 1.5) | | <0.001* | | | 2.07 | 0.13 | | | | (2.3, 1.8) | | | | <0.001* | | | 2.04 | | 0.13 | (2.3, 1.8) | | | <0.001* | |  |  |
| *Level 1 (individual)* | | | |  |  |  |  |  |  | |  | |  | |  | | |  |  | | | |  | | | |  | | |  | |  |  | | |  | |  |  |
| **Ownership of a functional latrine, γ_10_** | | | |  | -0.16 | 0.04 | (-0.1, -0.2) | <0.001* | -0.04 | | 0.06 | | (0.1, -0.2) | | 0.423 | | | -0.03 | 0.06 | | | | (0.1, -0.1) | | | | 0.546 | | | -0.04 | | 0.06 | (0.1, -0.2) | | | 0.452 | |  |  |
| **Sanitation Insecurity** |  |  |  |  |  |  |  |  |  | |  | |  | |  | | |  |  | | | |  | | | |  | | |  | |  |  | | |  | |  |  |
| 1: Potential Harms, γ_20_ |  |  |  |  |  |  |  |  | 0.05 | | 0.04 | | (0.1, 0.0) | | 0.182 | | | 0.08 | 0.03 | | | | (0.1, 0.0) | | | | 0.019* | | | 0.08 | | 0.03 | (0.2, 0.0) | | | 0.020* | |  |  |
| 2: Social expectations & repercussions, γ_30_ | | | |  |  |  |  |  | -0.29 | | 0.05 | | (-0.2, -0.4) | | <0.001* | | | -0.24 | 0.05 | | | | (-0.1, -0.3) | | | | <0.001* | | | -0.24 | | 0.05 | (-0.1, -0.3) | | | <0.001* | |  |  |
| 3: Physical exertion or strain, γ_40_ | | | |  |  |  |  |  | 0.51 | | 0.07 | | (0.6, 0.4) | | <0.001* | | | 0.59 | 0.06 | | | | (0.7, 0.5) | | | | <0.001* | | | 0.59 | | 0.06 | (0.7, 0.5) | | | <0.001* | |  |  |
| 4: Night Concerns, γ_50_ |  |  |  |  |  |  |  |  | 0.13 | | 0.02 | | (0.2, 0.1) | | <0.001* | | | 0.17 | 0.02 | | | | (0.2, 0.1) | | | | <0.001* | | | 0.17 | | 0.02 | (0.2, 0.1) | | | <0.001* | |  |  |
| 5: Dependent support, γ_60_ | | |  |  |  |  |  |  | 0.00 | | 0.04 | | (0.1, -0.1) | | 0.939 | | | -0.01 | 0.04 | | | | (0.1, -0.1) | | | | 0.886 | | | -0.01 | | 0.04 | (0.1, -0.1) | | | 0.885 | |  |  |
| 6: Physical agility, γ_70_ |  |  |  |  |  |  |  |  | 0.15 | | 0.02 | | (0.2, 0.1) | | <0.001* | | | 0.01 | 0.02 | | | | (0.1, 0.0) | | | | 0.825 | | | 0.00 | | 0.02 | (0.1, 0.0) | | | 0.862 | |  |  |
| 7: Defecation place, γ_80_ | | | |  |  |  |  |  | 0.04 | | 0.04 | | (0.1, 0.0) | | 0.285 | | | 0.03 | 0.04 | | | | (0.1, 0.0) | | | | 0.480 | | | 0.03 | | 0.04 | (0.1, 0.0) | | | 0.404 | |  |  |
| **Life Stage (Stage 1: Unmarried as referent)** | | | |  |  |  |  |  |  | |  | |  | |  | | |  |  | | | |  | | | |  | | |  | |  |  | | |  | |  |  |
| Stage 2: Recently Married, γ_90_ | | | |  |  |  |  |  |  | |  | |  | |  | | | 0.15 | 0.05 | | | | (0.2, 0.1) | | | | 0.001* | | | 0.15 | | 0.05 | (0.2, 0.1) | | | 0.001* | |  |  |
| Stage 3: Married over 3 years, γ_100_ | | | |  |  |  |  |  |  | |  | |  | |  | | | 0.14 | 0.04 | | | | (0.2, 0.1) | | | | <0.001* | | | 0.14 | | 0.04 | (0.2, 0.1) | | | <0.001* | |  |  |
| Stage 4: Over 49 years old, γ_110_ | | | |  |  |  |  |  |  | |  | |  | |  | | | 0.34 | 0.05 | | | | (0.4, 0.2) | | | | <0.001* | | | 0.34 | | 0.05 | (0.5, 0.2) | | | <0.001* | |  |  |
| **Water access within dwelling / compound, γ_120_** | | | |  |  |  |  |  |  | |  | |  | |  | | | 0.00 | 0.04 | | | | (0.1, -0.1) | | | | 0.989 | | | 0.00 | | 0.04 | (0.1, -0.1) | | | 0.946 | |  |  |
| **Bathing Area within dwelling / compound, γ_130_** | | | |  |  |  |  |  |  | |  | |  | |  | | | -0.07 | 0.05 | | | | (0.0, -0.2) | | | | 0.218 | | | -0.06 | | 0.05 | (0.1, -0.2) | | | 0.267 | |  |  |
| **Possession of 'BPL' card, γ_140_** | | | |  |  |  |  |  |  | |  | |  | |  | | | 0.04 | 0.03 | | | | (0.1, 0.0) | | | | 0.189 | | | 0.04 | | 0.03 | (0.1, 0.0) | | | 0.192 | |  |  |
| **No current illness, γ_150_** | | | |  |  |  |  |  |  | |  | |  | |  | | | -0.24 | 0.04 | | | | (-0.2, -0.3) | | | | <0.001* | | | -0.24 | | 0.04 | (-0.2, -0.3) | | | <0.001* | |  |  |
| **Social Support, γ_160_** |  |  |  |  |  |  |  |  |  | |  | |  | |  | | | -0.06 | 0.02 | | | | (0.0, -0.1) | | | | <0.001* | | | -0.06 | | 0.02 | (0.0, -0.1) | | | <0.001* | |  |  |
| *Level 2 (community)* |  |  |  |  |  |  |  |  |  | |  | |  | |  | | |  |  | | | |  | | | |  | | |  | |  |  | | |  | |  |  |
| **Intervention Status, γ_01_** | | |  |  |  |  |  |  |  | |  | |  | |  | | |  |  | | | |  | | | |  | | | 0.07 | | 0.07 | (0.2, -0.1) | | | 0.162 | |  |  |
|  | **Random Parameters** | | | | | | | | | | | | | | | | | | | | | | | | | | | | | | | | | | | | |  |  |
|  | *Variance Component, Standard Deviation, p-value* | | | | | | | | | | | | | | | | | | | | | | | | | | | | | | | | | | | | |  |  |
| Intercept, u0 | 0.03 | 0.2 | <0.001* |  | 0.03 | 0.1 | <0.001* |  | 0.02 | | 0.1 | | <0.001* | |  | | | 0.02 | 0.1 | | | | <0.001* | | | |  | | | 0.01 | | 0.1 | <0.001* | | |  | |  |  |
| Level-1, *r* | 0.29 | 0.6 |  |  | 0.29 | 0.6 |  |  | 0.23 | | 0.5 | |  | |  | | | 0.21 | 0.5 | | | |  | | | |  | | | 0.21 | | 0.5 |  | | |  | |  |  |
|  | **Additional Model Components** | | | | | | | | | | | | | | | | | | | | | | | | | | | | | | | | | | | | |  |  |
| ICC | 0.1 | |  |  |  |  |  |  |  | |  | |  | |  | | |  |  | | | |  | | | |  | | |  | |  |  | | |  | |  |  |
| Deviance | 2235.2 | |  |  | 2212.9 | |  |  | 2256.5 | | | |  | |  | | | 1758.5 | | | | |  | | | |  | | | 1756.0 | | |  | | |  | |  |  |
| # Estimated Parameters | 3.0 | |  |  | 4.0 | |  |  | 11.0 | | | |  | |  | | | 19.0 | | | |  | | | | |  | | | 20.0 | | |  | | |  | |  |  |
| Variance Reduction,τ00 |  |  |  |  | 0.0 | |  |  | 0.4 | | | |  | |  | | | 0.0 | | | |  | | | | |  | | | 0.1 | | |  | | |  | |  |  |
| Variance Reduction, ᵟ^2^ |  |  |  |  | 0.0 | |  |  | 0.2 | | | |  | |  | | | 0.1 | | | |  | | | | |  | | | 0.0 | | |  | | |  | |  |  |
| AIC |  | |  |  | -2204.9 | |  |  | -1893.9 | | | |  | |  | | | -1720.5 | | |  | | | | |  | | | -1716.0 | | | |  | | |  | |  |  |
| BIC |  | |  |  | -2184.6 | |  |  | -1836.6 | | | |  | |  | | | -1621.6 | | |  | | | | |  | | | -1611.9 | | | |  | | |  | |  |  |
| *Significant at p <0.05 |  |  |  |  |  |  |  |  | |  | |  | |  | |  | | | |  | | | |  |  | | | |  | |  | | |  |  | | |  | |
